# Closed multilumen infusion system reduces CLABSI and antibiotic exposure in very preterm neonates

**DOI:** 10.1136/bmjpo-2026-005071

**Published:** 2026-10-20

**Authors:** Fabrizio Maria Dacquino, Julien Gotchac, Eloïse Le Cabec, Emilie Pauquet, Rémy Gérard

**Affiliations:** 1 Department of Neonatal Intensive Care Unit, University Hospital Centre Bordeaux, Bordeaux, France; 2 Department of Pediatric Intensive Care Unit, University Hospital Centre Bordeaux, Bordeaux, France; 3 IHU Liryc Institut des Maladies du Rythme Cardiaque, Pessac, France; 4 ImmunoConcEpT, CNRS UMR 5164, Inserm ERL 1303, Universite de Bordeaux, Bordeaux, France

**Keywords:** Neonatology, Intensive Care Units, Neonatal

## Abstract

**Background:**

Central line-associated bloodstream infections (CLABSIs) remain a major morbidity in preterm neonates despite infection prevention bundles. While a closed multilumen infusion system (CMLIS) previously demonstrated CLABSI reduction, validation across different clinical settings remains limited.

**Objective:**

To evaluate whether CMLIS implementation in a different neonatal intensive care unit (NICU) confirms reduced CLABSI incidence density and optimises antimicrobial stewardship.

**Methods:**

This retrospective study in a French tertiary NICU included preterm neonates (23^+0^ to 31^+6^ weeks’ gestation) admitted between January 2024 and October 2025. Using multivariable Poisson regression and propensity score matching (PSM), we compared infants managed with standard manifolds versus the CMLIS. The primary outcome was CLABSI incidence density per 1000 catheter-days (defined as a central catheter-associated positive blood culture with infection signs, treated with appropriate antibiotics for ≥5 days). Secondary outcomes included antibiotic days of therapy, mortality, late-onset sepsis, prematurity-related complications and NICU length of stay.

**Results:**

Among the 170 neonates included, CMLIS implementation was associated with a significant reduction in the primary outcome (adjusted incidence rate ratio (aIRR): 0.50; p=0.025). This protective effect was confirmed in the PSM cohort (IRR: 0.55; p=0.03). Regarding antimicrobial stewardship, total antibiotic consumption decreased by 41% (aIRR: 0.59; p=0.002) and vancomycin usage decreased by 39% (aIRR: 0.61; p=0.006). No significant differences were observed in other clinical outcomes or exploratory mortality.

**Conclusions:**

CMLIS implementation was associated with lower CLABSI incidence density and reduced antibiotic exposure. These findings suggest CMLIS offers valuable incremental benefits when baseline hygiene bundles are optimised, though further prospective validation is warranted.

**WHAT IS ALREADY KNOWN ON THIS TOPIC**

- While standard multilumen infusion systems are associated with increased central line-associated bloodstream infections (CLABSI) incidence in adults,^31^ data in the fragile neonatal population remain scarce, with only preliminary evidence suggesting that closed multilumen infusion system (CMLIS) mitigates this risk.^3^

**WHAT THIS STUDY ADDS**

- By observing an independent neonatal intensive care unit setting, our study corroborates earlier findings, showing that CMLIS implementation is associated with decreased CLABSI incidence density and a corresponding reduction in antibiotic exposure.

**HOW THIS STUDY MIGHT AFFECT RESEARCH, PRACTICE OR POLICY**

- CMLIS could complement standard infection prevention bundles to reduce infectious risks, limit antibiotic exposure and lessen parental anxiety, while allowing up to 21 days of use compared with the 7-day limit of standard systems. However, prospective, multicentre randomised controlled trials are strictly required to definitively validate these retrospective findings before universal adoption.

## Introduction

 Very preterm neonates depend on prolonged central venous access for life-saving parenteral nutrition and complex polytherapy.^1 2^ However, the medical devices essential for survival expose this vulnerable population to a disproportionate risk of late-onset sepsis (LOS) and central line-associated bloodstream infections (CLABSI).^3^ Despite significant advances in neonatal care, LOS continues to represent a major cause of preventable morbidity and mortality in preterm infants. Large cohort studies report an incidence of approximately 21% among infants born before 28 weeks’ gestation.^4 5^ Crucially, three out of four LOS cases in this extremely preterm population are CLABSI episodes.^5^ Furthermore, data from the French national Néocat registry underscore that this infectious burden increases as birth weight and gestational age decrease: for non-umbilical central lines, baseline CLABSI incidence densities characteristically scale from approximately 8 per 1000 catheter-days in infants weighing 1001–1500 g, up to more than 22 per 1000 catheter-days in the most fragile subset weighing 750 g or less.^6^

Beyond the heightened risk of acute mortality, these infections are independently associated with severe long-term complications, including bronchopulmonary dysplasia (BPD), retinopathy of prematurity (ROP) and neurodevelopmental impairment,^7 8^ while simultaneously disrupting the developing microbiome and accelerating antimicrobial resistance. Collectively, this profound clinical and ecological burden underscores the critical and urgent need for comprehensive, wide-ranging preventative strategies.^9 10^

To mitigate these risks, contemporary neonatal practice relies on evidence-based infection prevention frameworks, including standardised insertion and maintenance bundles, dedicated vascular access teams, and meticulous hub disinfection protocols. Furthermore, technical innovations such as cyanoacrylate tissue adhesive have been introduced to improve catheter securement and provide a physical barrier against extraluminal bacterial migration.

Nonetheless, while these practices have systematically reduced extraluminal contamination, the intraluminal pathway remains highly vulnerable. This is because traditional multiinfusion configurations, ranging from simple manifolds to complex multilumen extension lines, universally function as open systems requiring frequent manual assembly and fluid track manipulations inside the incubator. This open architecture represents a primary vulnerability, exposing the vascular access hub to environmental pathogens and contradicting the neuroprotective principle of minimal handling.^11^ The closed multiline infusion system addresses this vulnerability by introducing a preassembled, closed design that fundamentally shifts line management and connection consolidation outside the incubator environment and allows for up to 21 days of extended use, unlike traditional open devices. An initial single-centre study, carried out in Lyon, France, which is currently the only setting where closed multilumen infusion system (CMLIS) data have been reported, suggested an association between reduction in infection rates and CMLIS implementation.^3^ Consequently, the reproducibility of these findings in other neonatal units remains to be determined.

Therefore, the primary objective of this study was to evaluate whether previously reported CMLIS benefits could be replicated when adjusting for baseline neonatal severity in a different neonatal unit. While randomised controlled trials are the gold standard for establishing causality, we mitigated the inherent limitations of our before-and-after design using multivariable regression and propensity score matching (PSM).

## Methods

### Patients and settings

This observational, retrospective and comparative single-centre study was conducted at the tertiary neonatal intensive care unit (NICU) of the University Hospital Pellegrin in Bordeaux, France.

The study population included preterm neonates with gestational ages between 23^+^⁰ and 31^+^⁶ weeks admitted between January 2024 and October 2025. Eligibility required admission within the first 24 hours of life, the placement of a neonatal peripherally inserted central catheter (n-PICC), and survival beyond the initial 72 hours of life. Exclusion criteria included major congenital malformations incompatible with life, suspected or confirmed congenital immunodeficiencies, and missing or incomplete medical records.

The study compared two historical cohorts defined by the timing of admission and the infusion technology employed. The control group consisted of patients admitted during 2024 managed with a standard infusion manifold, while the intervention group included patients admitted during 2025 managed with a CMLIS. All patients were followed until reaching 36 weeks of corrected gestational age.

The implementation of the CMLIS was nested within a pre-existing, standardised quality-improvement framework for central line management to target infusion system complexity and reduce intraincubator manipulations, rather than to substitute for fundamental infection control. This baseline infrastructure encompassed an institutional insertion bundle (maximum sterile barriers, chlorhexidine antisepsis), a maintenance bundle (daily dressing integrity checks, standardised catheter securement evaluation, and a strict 20 s ‘hub scrub’ protocol before any line access), and regular concurrent catheter care audits performed by senior nursing staff. To ensure high clinical adherence, a comprehensive multidisciplinary training programme, targeting attending neonatologists, paediatric residents, staff nurses and nursing students, was launched 3 months prior to the device clinical rollout, focusing strictly on aseptic management, line consolidation and proper device handling.

Across both periods (before and after CMLIS implementation), the infants were managed using 2 Fr polyurethane n-PICC. In the CMLIS group, the CMLIS used was the Edelvaiss Multiline-Néo (Doran International, Toussieu, France). Furthermore, the standard nursing workload for intensive care beds was maintained at a strict 1:2 nurse-to-patient ratio, supplemented by one dedicated support nurse for every 14 beds to ensure baseline care stability. Structurally, our NICU comprises 14 intensive care beds, housed in single-patient rooms, with the exception of two double rooms and 10 subintensive care beds. Our unit accounts for approximately 200 admissions per year.

Clinical data were extracted from the IntelliSpace Critical Care and Anaesthesia, Philips electronic medical records and unit admission registries. To ensure patient privacy and confidentiality, all data were managed using an anonymised and password-protected dataset.

### Outcomes

The primary outcome of this study was the incidence density of CLABSI, expressed as the number of episodes per 1000 catheter-days. CLABSI was defined based on established Centers for Disease Control and Prevention/National Healthcare Safety Network (CDC/NHSN) criteria tailored to the neonatal population,^11 12^ with an additional study-specific requirement. Standard criteria required the isolation of a recognised pathogen from at least one blood culture, accompanied by clinical and/or laboratory signs of infection, in the presence of an indwelling central venous catheter (CVC) (or within 48 hours of its removal). To optimise specificity and exclude transient contamination or asymptomatic bacteraemia, we applied a study-specific modification restricting our primary outcome to episodes where appropriate targeted antibiotic therapy was initiated and maintained for a minimum of five consecutive days.

Secondary outcomes included overall mortality, LOS, antimicrobial exposure, NICU length of stay (defined as the duration of admission within the tertiary NICU), and major prematurity-related morbidities. LOS was defined as a systemic infection occurring after 72 hours of life, requiring new clinical signs coupled with altered inflammatory biomarkers prompting more than 5 days of systemic antibiotic therapy. Antimicrobial exposure was evaluated using the standardised days of therapy (DOT) metric, encompassing all systemic intravenous and oral antibiotics administered during the entire NICU hospitalisation; empirical treatments initiated within the first 48 hours of life for suspected early-onset sepsis were excluded to avoid baseline screening bias. Major prematurity-related morbidities evaluated included BPD at 36 weeks,^13^ ROP greater than stage 2,^14^ necrotising enterocolitis (NEC) greater than stage 2,^15^ neurological complications, specifically intraventricular haemorrhage (IVH) greater than grade 2^16 17^ and periventricular leukomalacia.

### Sample size calculation

Due to the retrospective nature of the study, a convenience sample of all consecutive eligible neonates admitted to the NICU between January 2024 and October 2025 was included, resulting in a final cohort of 170 patients (89 control; 81 CMLIS). Because the sample size was predetermined by annual admission rates rather than driven by a prospective recruitment target, a post hoc calculation was performed to estimate the available power under a Poisson framework. Based on a total exposure of 3252 catheter-days, a baseline historical CLABSI rate of 15.38 per 1000 catheter-days, and a two-sided alpha of 0.05, this available sample size provided greater than 80% power to detect an incidence rate ratio (IRR) ≤0.50 in the CMLIS group. However, this statistical power estimation does not account for potential temporal confounding, such as concurrent practice changes or seasonal effects, inherent to comparing historical (2024) and contemporary (2025) cohorts.

### Statistical analysis

Descriptive statistics were used to summarise the clinical characteristics of the study population. Categorical variables were presented as absolute frequencies and percentages and compared using the two-sided Fisher’s exact test. Continuous variables, characterised by a non-normal distribution, were presented as medians and IQRs and compared using either the Mann-Whitney U test or the two-sample Wilcoxon rank-sum test. Additionally, comparisons of incidence density rates were performed using the Poisson exact test.

The association between CMLIS implementation and CLABSI incidence density and secondary outcomes (total antibiotic DOT and vancomycin DOT) was evaluated using Poisson regression models.^17 18^ To calculate adjusted IRRs (aIRRs) and their corresponding 95% CIs, the duration of catheter days was defined as the exposure variable.

In accordance with international reporting guidelines,^19 20^ both relative and absolute measures of effect were calculated. The aIRR was used to assess the strength of association, while the adjusted Incidence Rate Difference (aIRD) was reported to quantify the absolute clinical impact. Furthermore, adjusted predictive margins were estimated to compare absolute outcomes between the control and CMLIS groups, holding clinical confounders, including gestational age, SNAPPE-II (*Score for Neonatal Acute Physiology with Perinatal Extension-II*) score, FGR and invasive ventilation, at their mean values.

The distribution of the NICU length of stay (NLOS) was assessed for normality using the Skewness/Kurtosis test. Given the non-significant skewness, the association of CMLIS with NLOS was evaluated using multivariate linear regression. For major prematurity-related morbidities, multivariable logistic regression was used to calculate OR.

Covariates were selected a priori based on clinical relevance, previously identified risk factors for neonatal CLABSI, and factors potentially associated with both treatment allocation and infectious outcomes. To avoid model overfitting given the limited number of infectious events and according to the objective of each analytical approach, multivariable regression models were adjusted using a parsimonious set of clinically relevant confounders such as gestational age, FGR, invasive mechanical ventilation lasting more than 48 hours, and the SNAPPE-II score. As an exploratory outcome, the association between the CMLIS and mortality occurring between 72 hours of life and 36 weeks of corrected gestational age was evaluated using a multivariable logistic regression model. Due to the limited number of events observed during the study period, this exploratory model was adjusted for gestational age only to avoid model overfitting.

To further validate outcomes that reached statistical significance in the primary multivariate analysis, a PSM analysis was performed as a sensitivity analysis. Neonates were matched 1:1 using a nearest-neighbour algorithm with a 0.20 calliper. In accordance with international literature,^20–23^ the matching process incorporated the same set of baseline covariates potentially associated with treatment allocation in order to optimise covariate balance and improve exchangeability between groups. After matching, Poisson regression analysis for CLABSI incidence density, total antibiotics DOT and vancomycin DOT was repeated within the balanced cohort to estimate the treatment effect. Robust SEs were used to ensure the reliability of the estimates and to account for the paired nature of the propensity score-matched cohort.

All statistical analyses were conducted using STATA V.13 (StataCorp). Statistical significance was set at p<0.05.

### Patient and public involvement

Patients and the public were not involved in the design, conduct, reporting or dissemination plans of this research. This study involved a retrospective analysis of clinical data collected during routine hospital care; therefore, no patient or public involvement was feasible or sought in the research process.

## Results

### Study population and baseline characteristics

A total of 330 newborns were admitted to the NICU during the study period. After excluding 160 infants, as shown in figure 1, the final study population consisted of 170 neonates. This cohort included 89 infants in the control group (2024) and 81 in the CMLIS group (2025).

**Figure 1:**
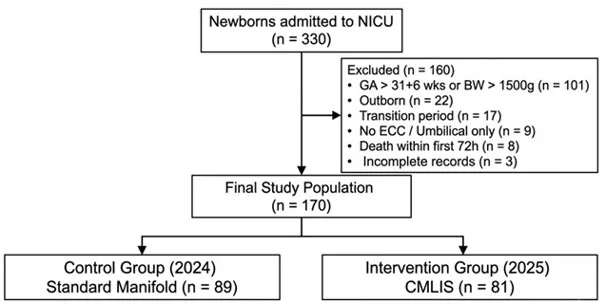
BW, birth weight; CMLIS, closed multilumen infusion system; ECC, epicutaneous-cavous catheter; GA, gestational age; NICU, neonatal intensive care unit

The baseline demographic characteristics, clinical features and main neonatal morbidities of the study population across the two study periods are summarised in table 1. The study population primarily consisted of very preterm infants, frequently presenting with respiratory distress syndrome and elevated SNAPPE-II scores at admission.

**Table 1:** Baseline demographic, clinical features and main neonatal morbidities in the overall cohort

	All N=170 (%)	Control n=89 (%)	CMLIS n=81 (%)	P value
Gestational age, median (IQR), weeks	28 (26.7, 29.4)	28.4 (26.7, 29.4)	27.8 (26.7, 29.4)	0.31†
Birth weight, median (IQR), g	1000 (840, 1180)	1010 (840, 1180)	990 (820, 1170)	0.57†
Male sex	82 (48)	41 (51)	41 (46)	0.64*
FGR (<3rd)	15 (9)	4 (5)	11 (12)	0.10*
Complete antenatal corticosteroids	137 (80)	66 (81)	71 (80)	0.84*
SNAPPE-II score, median (IQR)	18 (5, 31)	16 (5, 31)	22 (7, 30)	0.24†
Invasive ventilation (>48 hours)	60 (35)	31 (38)	29 (33)	0.52*
Haemodynamic support (>48 hours)	60 (35)	30 (37)	30 (34)	0.74*
CLABSI density/1000 catheter days	11.68	15.38	8.27	0.06§
Late-onset sepsis episodes	50 (29.4)	27 (30.3)	23 (28.4)	0.86*
Catheter days, median (IQR), days	12 (8, 24)	12 (8, 21)	11 (7, 28)	0.53†
CUR, median (IQR)	0.30 (0.18, 0.63)	0.33 (0.19, 0.60)	0.28 (0.18, 0.63)	0.80‡
PN length, median (IQR), days	13 (9, 25)	13 (9, 22)	12 (8, 31)	0.63‡
NEC >2	14 (8.2)	5 (5.6)	9 (11.1)	0.26*
ROP >2	52 (30.5)	25 (28.1)	27 (33.3)	0.50*
IVH >2	9 (5.2)	6 (6.7)	3 (3.7)	0.50*
BPD	58 (34.1)	26 (29.2)	32 (39.5)	0.19*

* P values were calculated using the two-sided Fisher’s exact test.

† p values were calculated using the Mann-Whitney U test.

‡ p values were calculated using the two-sample Wilcoxon rank-sum (Mann-Whitney U) test.

§ p values were calculated using the Poisson exact test.

BPD, bronchopulmonary dysplasia; CLABSI, central line-associated bloodstream infection; CMLIS, closed multilumen infusion system; CUR, catheter utilisation ratio; FGR, fetal growth restriction; IVH, intraventricular haemorrhage; NEC, necrotising enterocolitis; PN, parenteral nutrition; ROP, retinopathy of prematurity; SNAPPE-II, Score for Neonatal Acute Physiology with Perinatal Extension-II.

### Multivariable analysis

In the baseline control group, the overall crude CLABSI density rate was 15.38 episodes per 1000 catheter-days. Stratified by birth weight, baseline rates were 20.8 episodes per 1000 catheter-days for infants ≤750 g, 17.8 for 751–1000 g and 8.7 for 1001–1500 g. Following CMLIS implementation, the crude CLABSI density rate decreased to 8.27 episodes per 1000 catheter-days in the intervention group, representing an unadjusted reduction of 46.2%.

As shown in table 2, after controlling for gestational age, fetal growth restriction, invasive mechanical ventilation and SNAPPE-II scores in the multivariable Poisson regression model, the implementation of the CMLIS was associated with an aIRR of 0.50 (95% CI 0.27 to 0.91; p=0.025). This clinical improvement was further supported by an aIRD of 6.5 episodes per 1000 catheter-days (95% CI −11.0 to −1.0; p=0.015).

**Table 2:** Multivariable Poisson regression analysis of risk factors for CLABSI and of total antibiotic exposure and vancomycin exposure (days of therapy per 1000 catheter-days)

		aIRR	95% CI	P value
CLABSI	CMLIS	0.50	0.27 to 0.91	0.025
	Gestational age	0.79	0.65 to 0.97	0.023
	SNAPPE II score	0.99	0.97 to 1.01	0.402
	FGR	2.05	0.73 to 5.75	0.170
	Mechanical ventilation	1.11	0.52 to 2.38	0.786
Antibiotics DOT	CMLIS	0.59	0.42 to 0.83	0.002
	Gestational age	0.85	0.75 to 0.96	0.011
	SNAPPE II score	1.00	0.99 to 1.01	0.944
	FGR	1.68	0.96 to 2.94	0.066
	Mechanical ventilation	1.75	1.14 to 2.70	0.011
Vancomycin DOT	CMLIS	0.61	0.43 to 0.87	0.006
	Gestational age	0.86	0.75 to 0.97	0.019
	SNAPPE II score	1.00	0.99 to 1.01	0.735
	FGR	1.81	1.02 to 3.22	0.043
	Mechanical ventilation	1.92	1.20 to 3.08	0.007

Results are presented as adjusted incidence rate ratio using catheter-days as the offset variable.

aIRR, adjusted incidence rate ratio; CLABSI, central line-associated bloodstream infection; CMLIS, closed multilumen infusion set; DOT, days of therapy; FGR, fetal growth restriction; SNAPPE II, Score for Neonatal Acute Physiology with Perinatal Extension-II.

Regarding pathogen distribution from positive blood cultures, CLABSI episodes in the control group were caused by *E. coli* (n=2), with the remaining cases attributed to coagulase-negative staphylococci (CoNS). In the CMLIS group, identified pathogens included *S. aureus* (n=1), *E. faecalis* (n=1) and CoNS for all remaining episodes.

Parallel to the reduction in infections, CMLIS was associated with a lower antimicrobial exposure. In the adjusted multivariable model, the implementation of CMLIS was associated with a significant 41% reduction in antibiotic use (aIRR 0.59; p=0.002), with an aIRD of −314 DOT per 1000 catheter-days (p=0.001). Similarly, vancomycin-specific therapy was reduced by 39% (aIRR 0.61; p=0.006), showing an aIRD of −110 DOT per 1000 catheter-days (p=0.003). Clinically, these absolute differences translated into a substantial saving of approximately 6 total antibiotic days and 2 vancomycin days for every neonate managed with the CMLIS. No reduction in piperacillin-tazobactam was seen (aIRR 1.01; p=0.95).

Detailed adjusted rates are summarised in table 3.

**Table 3:** Clinical impact and absolute measures of effect of the CMLIS intervention

	Adjusted rate (control)*	Adjusted rate (CMLIS)*	aIRD† (95% CI)	P value
CLABSI	12.9	6.4	−6.5 (−11 to −1)	0.015
Antibiotics DOT	675	402	−314 (−490 to −120)	0.001
Vancomycin DOT	246	151	−111 (−180 to −40)	0.003

CLABSI rates and antibiotic DOT (total and vancomycin) are expressed per 1000 catheter-days.

* Adjusted predictive margins: values represent the predicted incidence rates derived from multivariable Poisson regression models. Margins were calculated by holding all clinical covariates (gestational age, SNAPPE-II score, FGR and invasive mechanical ventilation) at their mean values, effectively isolating the treatment effect of the intervention.

† aIRD: represents the absolute difference in adjusted rates between the CMLIS and control groups.

aIRD, adjusted incidence rate difference; CLABSI, central line associated bloodstream infection; CMLIS, closed multilumen infusion set; DOT, days of therapy; FGR, fetal growth restriction; SNAPPE-II, Score for Neonatal Acute Physiology with Perinatal Extension-II.

The overall mortality rate was 8.2% (14 episodes over 170 patients). In the exploratory multivariable logistic regression model adjusted for gestational age, the use of the CMLIS was associated with an adjusted OR (aOR) for death of 0.74 (95% CI 0.24 to 2.27; p=0.599). The causes of death across the entire cohort were distributed as follows: five cases were secondary to severe IVH, four to severe persistent pulmonary hypertension of the newborn, and four to NEC; for one patient, specific data regarding the cause of death were unavailable. No deaths were clinically attributed to septic shock or bloodstream infections. The use of CMLIS was not associated with NICU length of stay (Coefficient: 0.62; 95% CI −4.27 to 5.52; p=0.80). No significant differences were observed between groups regarding neither the LOS rate nor the incidence of prematurity-related complications.

### Propensity score matching

To corroborate the findings of the multivariable models, a PSM procedure was performed using a Probit model to pair neonates based on baseline covariates. The matching procedure resulted in a balanced cohort of 156 patients (78 pairs), effectively reducing the global mean bias from 17.2% to 5.9%, as shown in online supplemental figure S1, and eliminating baseline imbalances between the two groups, as reported in online supplemental table S1.

Within the PSM cohort, the implementation of CMLIS remained significantly associated with a reduction in CLABSI incidence density compared with the control group (IRR 0.55; 95% CI 0.32 to 0.94; p=0.03) (table 4).

**Table 4:** Poisson regression analysis of major outcomes in the PSM population

	Incidence rate ratio	95% CI	P value
CLABSI	0.55	0.32 to 0.94	0.030
Antibiotics DOT	0.71	0.50 to 0.99	0.044
Vancomycin DOT	0.71	0.50 to 1.02	0.061

All rates are expressed per 1000 catheter-day.

CLABSI, central line-associated bloodstream infection; DOT, days of therapy; PSM, propensity score matching.

Regarding antimicrobial exposure, the use of the CMLIS was associated with a significant reduction in overall therapy. After adjusting for catheter-days, the CMLIS group showed a 29% reduction in antibiotic incidence density (IRR 0.71; 95% CI 0.50 to 0.99; p=0.04). Focusing on vancomycin, the CMLIS group demonstrated a 29% marginally non-significant reduction in vancomycin usage (IRR 0.71; 95% CI 0.50 to 1.02; p=0.06).

## Discussion

In our population of 170 preterm neonates, the use of the CMLIS was associated with a decrease in both CLABSI incidence density and antimicrobial exposure. Specifically, the analysis showed an aIRD of −6.5 CLABSI episodes per 1000 catheter-days.

In our institution, the CMLIS was integrated into a comprehensive, pre-existing, multicomponent CLABSI prevention programme, which included standardised insertion and maintenance bundles, a dedicated vascular access team, strict catheter maintenance protocols and the use of cyanoacrylate tissue adhesive as an adjunct for catheter stabilisation and sealing. Within this framework, the CMLIS was introduced to provide an incremental benefit rather than a standalone solution, in line with current evidence emphasising multicomponent infection control.^23 24^

Prior to this intervention, despite the optimisation of these primary, evidence-based measures, our baseline unstratified CLABSI density rate (approximately 15.32 per 1000 catheter-days) appeared higher than general benchmarks, reflecting a significant case-mix bias. Stratified by birth weight, however, our baseline rates (20.8 for ≤750 g, 17.8 for 751–1000 g and 8.7 for 1001–1500 g) were comparable to national data from the French Néocat registry for non-umbilical CVCs (22.0, 17.2 and 8.4, respectively).^6^

The observed reduction in infections is likely related to the structural design of the CMLIS. By combining multiple pathways into a single, preassembled closed circuit, the system minimises pathogen entry points during routine connection and disconnection.^3^ In our study, the circuit remained in place safely for up to 21 days, which is longer than the traditional 7-day limit for open systems. Additionally, placing the infusion hub entirely outside the incubator environment may reduce environmental stress and sleep fragmentation for these fragile infants.^10 11^

Our observed CLABSI incidence density reduction was more modest than that reported in the pioneering Lyon cohort,^3^ likely because our use of multivariable adjustments and PSM yielded more conservative, epidemiologically realistic effect sizes or potentially due to variations in baseline healthcare-associated infection prevention protocols between the two institutions.

Microbiologically, CoNS were the predominant pathogens across both periods, aligning with typical neonatal CLABSI aetiology. The reduction in these device-associated infections likely accounts for the observed 41% decrease in vancomycin DOT (saving more than 110 days per 1000 catheter-days). As vancomycin is a primary therapy for CoNS in our unit, this trend suggests a potential benefit for antimicrobial stewardship. Ultimately, decreasing vancomycin exposure may help mitigate nephrotoxicity risks,^24^ limit multidrug-resistant selective pressure and preserve the gut microbiome,^25^ which could potentially lower the risk of intestinal dysbiosis and late-onset NEC.^26^ Conversely, the CMLIS had a negligible impact on NICU length of stay, severe prematurity-related complications or overall mortality. Our clinical review confirmed that these outcomes were exclusively driven by the severe non-infectious complexities of extreme prematurity rather than infectious events, highlighting the inherent clinical complexity of this fragile population.^27–29^

Beyond the direct clinical advantages, these findings open important exploratory considerations regarding healthcare resource allocation. Mitigating CLABSI episodes and averting prolonged intravenous antibiotic courses translate into substantial economic savings, confirming that the initial investment in closed infusion protocols can significantly curtail direct hospital expenditures.^30^

To ensure a fair comparison, baseline infection control practices included in our CLABSI prevention programme, as well as nursing staffing levels and nurse-to-patient ratios remained stable across both periods. Furthermore, from an implementation science perspective, the structured CMLIS training focused strictly on the technical operation of the new device, without altering general hygiene standards or clinical workflows.

Several limitations should be acknowledged.

First, the retrospective, before-and-after design cannot completely exclude the influence of temporal trends or changes in clinical practice over time. Although multivariable regression and PSM were used to balance the patient groups, these methods cannot eliminate residual confounding from unrecorded factors, such as subtle shifts in nursing compliance or patient turnover. Consequently, our results demonstrate a clinical association rather than direct causal inference.

Second, due to the historical and retrospective design, the study lacks granular, prospective data on day-to-day line management. We could not systematically measure the exact frequency of line manipulations, catheter accesses, hub contamination or incubator openings, nor could we track precise compliance rates for our concurrent preventive strategies. Therefore, while the CMLIS is structurally designed to minimise physical contact, the precise behavioural or microbiological mechanisms driving the CLABSI reduction remain hypothetical rather than definitively causal.

Third, our sample was determined by institutional volumes, meaning the study was not statistically powered to detect differences in secondary neonatal morbidities or outcomes like total NICU length of stay and overall mortality.

Lastly, due to blood volume constraints in extremely preterm infants, diagnoses relied on a single positive blood culture. Although applying CDC/NHSN criteria^12^with our study-specific 5-day antibiotic filter minimised misclassification from contamination, this introduces a structural limitation: it intrinsically links CLABSI classification (primary outcome) with antibiotic exposure (secondary outcome). Consequently, clinical decisions on treatment duration directly influenced case classification.

## Conclusions

Our findings suggest that a CMLIS may provide an adjunctive benefit once baseline hygiene and insertion bundles are optimised. These data align with the trends observed in previous studies,^3 30^ supporting further prospective evaluation of the CMLIS as an adjunct to established CLABSI-prevention and antimicrobial stewardship strategies. Consequently, prospective trials remain necessary to definitively establish its clinical efficacy and impact on long-term neonatal outcomes.

## Supplementary material

10.1136/bmjpo-2026-005071 online supplemental figure 1

10.1136/bmjpo-2026-005071 online supplemental table 1

## Data Availability

Data are available on reasonable request.
