# Supplementary material for: Closed multilumen infusion system reduces CLABSI and antibiotic exposure in very preterm neonates
Source: BMJ Paediatr Open. 2026 Oct 20;10(1):e005071. doi: 10.1136/bmjpo-2026-005071 (PMC13684196; doi:10.1136/bmjpo-2026-005071)
Supplement: Supplementary file 1 [file bmjpo-10-1-s002.pdf]

Closed Multi-Lumen Infusion System Reduces CLABSI and Antibiotic Exposure in Very Preterm Neonates

Supplementary Materials

Fabrizio Maria Dacquino MD[1], Julien Gotchac MD, PhD[2,3], Eloise Le Cabec, MD[1], Emilie Pauquet, MD[1], Rémy Gerard MD[1,4]

Affiliations:

[1] Neonatal Intensive Care Unit, University Hospital of Bordeaux, Bordeaux, France
[2] Pediatric Intensive Care Unit, Children's Hospital, Bordeaux University Hospital, Bordeaux, France
[3] IHU Liryc, INSERM 1045, University of Bordeaux, Bordeaux, France
[4] Univ. Bordeaux, ImmunoConcEpT, CNRS UMR 5164, Inserm ERL 1303, F-33000 Bordeaux, France

Corresponding author:

Dr Rémy Gerard

Neonatal Intensive Care Unit, University Hospital of Bordeaux, Bordeaux, France

Email: remy.gerard@chu-bordeaux.fr

Words count: abstract: 248; text: 2605.

Number of Tables and Figures: 4 tables; 1 figure

Cover title : Closed Multi-Lumen Infusion System Reduces CLABSI incidence density and Antibiotic Exposure in Very Preterm Neonates

Running head : CMLIS reduces CLABSI in preterm neonates

Data Availability Statement : The data that support the findings of this study are available from the corresponding author upon reasonable request.

Conflict of Interest statement : The authors declare no conflict of interest for this work.

Appendix S1

A Propensity Score Matching (PSM) analysis was performed to minimize selection bias between CMLIS neonates and controls. Propensity scores were estimated via multivariable logistic regression using four prespecified covariates: gestational age, fetal growth restriction, invasive mechanical ventilation (>48h), and baseline SNAPPE-II score. Patients were matched 1:1 (nearest-neighbor, without replacement) using a strict caliper of 0.20 standard deviations.

The initial unmatched cohort of 170 observations exhibited baseline imbalances (mean standardized bias 17.2%, Rubin's B 35.3, R^2 0.023). Matching generated a final balanced cohort of 156 patients (78 matched pairs). As shown in ***Figure S1***, the overall mean bias substantially decreased to 5.9%, and Rubin's B dropped to 13.3, falling well within acceptable limits. Furthermore, the model's pseudo R^2 decreased to 0.003, confirming the elimination of systematic differences.

At the individual covariate level (***Table S1***), absolute standardized differences (SMD) were reduced. Overall, the PSM successfully yielded two highly comparable groups, allowing for a robust and unbiased evaluation of the clinical outcomes.

Supplementary Materials

Figure S1 *Love plot of standardized mean differences before and after matching: circles represent the initial bias between the No CMLIS and the CMLIS cohorts, while crosses indicate the bias after one-to-one nearest-neighbor matching. The alignment of all variables toward the vertical zero line demonstrates that the procedure successfully eliminated systematic differences and achieved high covariate balance.* ***FGR****: fetal growth restriction.*

Table S1 *Post Propensity Score Matching demographic,clinical features, and main neonatal morbidities of neonates.*

	All N = 156	Control n = 78 (%)	CMLIS n = 78 (%)	*SMD*
Gestational age, median [IQR], weeks	28 [26.7, 29.4]	28.2 [26.5, 29.4]	27.8 [26.7, 29.4]	0.06
Birth weight, median [IQR], g	1005 [840, 1180]	1010 [840, 1180]	1000 [860, 1180]	0.04
Male sex	74 (47.4)	35 (44.8)	39 (50)	0.10
FGR (<3rd)	8 (5.1)	4 (5.1)	4 (5.1)	0.00
Complete Antenatal Corticosteroids	124 (79.4)	61 (78.2)	63 (80.7)	0.06
SNAPPE-II score, median [IQR]	17 [5, 30]	15 [5, 30]	22 [7, 30]	0.13
Invasive ventilation (>48h)	54 (34.6)	26 (33.3)	28 (35.9)	0.05
Hemodynamic support (>48h)	55 (35.26)	27 (34.6)	28 (35.9)	0.02
CLABSI density /1000 catheter days	12.89	17.11	9.06	0.43
Late-onset sepsis episodes	48 (30.7)	26 (33.3)	22 (28.2)	0.11
Catheter days, median [IQR], days	11 [7.5, 22]	12 [8, 21]	10.5 [7, 24]	0.10
CUR, median [IQR]	0.29 [0.18, 0.62]	0.33 [0.19, 0.60]	0.27 [0.18, 0.62]	0.02
PN length, median [IQR], days	13 [9, 24]	13 [9, 22]	11 [8, 27]	0.11
NEC >2	14 (8.9)	5 (6.4)	9 (11.5)	0.18
ROP >2	47 (30.1)	23 (29.4)	24 (30.7)	0.02
IVH >2	7 (4.4)	4 (5.1)	3 (3.8)	0.05
BPD	53 (33.9)	24 (30.7)	29 (37.1)	0.13

BPD: *bronchopulmonary dysplasia;* ***CLABSI:*** *central line-associated bloodstream infection;* ***CMLIS:*** *Closed Multi-Line Infusion System;* ***CUR:*** *catheter utilization ratio;* ***d:*** *days;* ***FGR:*** *fetal growth restriction;* ***g:*** *grams;* ***IVH:*** *intraventricular hemorrhage;* ***IQR:*** *interquartile range;* ***NEC:*** *necrotizing enterocolitis;* ***PN:*** *parenteral nutrition;* ***ROP:*** *retinopathy of prematurity;* ***SMD:*** *standardized mean difference;* ***SNAPPE-II:*** *Score for Neonatal Acute Physiology with Perinatal Extension-II.*

Values are expressed as n (%) or median [IQR] unless otherwise specified. Due to the paired nature of the propensity score-matched cohort, baseline balance between the groups was evaluated using the Standardized Mean Difference (SMD) rather than traditional p-values. An absolute SMD < 0.10 indicates an excellent covariate balance between the matched groups.
