# Supplementary material for: Closed multilumen infusion system reduces CLABSI and antibiotic exposure in very preterm neonates
Source: BMJ Paediatr Open. 2026 Oct 20;10(1):e005071. doi: 10.1136/bmjpo-2026-005071 (PMC13684196; doi:10.1136/bmjpo-2026-005071)
Supplement: Supplementary file 2 [file bmjpo-2026-005071.reviewer_comments.pdf]

PEER REVIEW HISTORY

BMJ Paediatrics Open publishes all reviews undertaken for accepted manuscripts. Reviewers are asked to complete a checklist review form and are provided with free text boxes to elaborate on their assessment. These free text comments are reproduced below.

ARTICLE DETAILS

**TITLE (PROVISIONAL)**	Closed Multi-Lumen Infusion System Reduces CLABSI and Antibiotic Exposure in Very Preterm Neonates
**AUTHORS**	Dacquino, Fabrizio Maria<br>Gotchac, Julien<br>Le Cabec, Eloïse<br>Pauquet, Emilie<br>Gérard, Rémy

VERSION 1 – REVIEW

**REVIEWER NAME**	van Rens, Roland
**REVIEWER AFFILIATION**	Hamad Medical Corporation, Neonatal Intensive Care Unit (NICU) Women's Wellness and Research Center
**REVIEWER CONFLICT OF INTEREST**	No Conflict of Interest
**AI DISCLOSURE**	No, I have not used AI
**DATE REVIEW RETURNED**	30-Jun-2026

**GENERAL COMMENTS**	Overall assessment<br>This manuscript addresses an important clinical question and reports an association between implementation of a closed multi-lumen infusion system (CMLIS) and reduced CLABSI incidence in a single tertiary NICU. However, I have significant concerns regarding the underlying scientific rationale, interpretation of the findings, and discussion.<br>The central premise of the study appears to be that changing the infusion system is the primary strategy to reduce CLABSI. However, CLABSI is a multifactorial complication, and contemporary prevention relies on a comprehensive quality improvement programme including standardized insertion and maintenance bundles, vascular access expertise, catheter securement, hub disinfection, staff competency, audit and feedback, and implementation science. The manuscript does not describe whether these established strategies were already fully implemented or whether the underlying causes of the relatively high baseline CLABSI rate had been systematically investigated.<br>In my opinion, this represents the principal weakness of the manuscript. Before evaluating an advanced technological intervention, it is essential to understand the causes of the existing infection burden and demonstrate that the remaining infections are likely attributable to the aspect of care targeted by the intervention. Without this context, the study cannot determine whether the observed improvement reflects the effect of the infusion system itself or broader changes in clinical practice.<br>For this reason, I believe the Discussion requires substantial revision and should begin by acknowledging this limitation.<br><br>Major comments

	1. Scientific rationale<br>The fundamental scientific rationale requires reconsideration.<br>The baseline CLABSI incidence reported in this study is 16.4 per 1,000 catheter-days, decreasing to 9.05 per 1,000 catheter-days following implementation of the CMLIS. Although this represents a reduction, the post-intervention rate remains relatively high, indicating that the underlying problem has not been resolved.<br>In quality improvement, advanced technological interventions are generally introduced after established infection prevention strategies have been optimized and residual causes of failure have been systematically investigated. The manuscript does not describe whether such an evaluation was undertaken.<br>Specifically, there is no information regarding:<br>• insertion bundle compliance<br>• maintenance bundle compliance<br>• vascular access team involvement<br>• catheter care audits<br>• hub disinfection compliance<br>• dressing integrity<br>• catheter securement<br>• root-cause analyses<br>• failure mode analysis<br>• concurrent quality-improvement initiatives<br>Without demonstrating that excessive infusion system manipulation was the dominant remaining contributor to CLABSI, it is difficult to conclude that the observed reduction is primarily attributable to the CMLIS.<br>I strongly recommend that the Discussion begins by acknowledging this limitation and positioning the intervention as one potential component of a comprehensive CLABSI prevention strategy rather than the principal solution.<br><br>2. Study design and interpretation<br>The Introduction appropriately critiques previous observational before-and-after studies for their susceptibility to temporal bias. However, the present investigation employs the same underlying historical cohort design, although strengthened by multivariable adjustment and propensity score matching.<br>These statistical methods improve adjustment for measured confounders but cannot eliminate residual confounding or secular trends. The manuscript should therefore avoid implying causal inference.<br><br>3. Study objective<br>The objective is clearly presented in the Abstract but is not explicitly stated at the end of the Introduction. A concise statement of the research question would improve the manuscript structure.<br><br>4. Background<br>The Introduction does not adequately position the intervention within current neonatal vascular access practice.<br>Evidence-based strategies including insertion and maintenance bundles, vascular access teams, standardized catheter care, catheter securement, hub disinfection, audit programmes, implementation science, and cyanoacrylate tissue adhesive should be discussed before introducing the CMLIS.<br><br>5. Mechanism<br>The Discussion repeatedly proposes that reduced manipulation led to reduced contamination and fewer infections.

	However, the study did not measure:<br>• manipulation frequency<br>• catheter accesses<br>• hub contamination<br>• incubator openings<br>• nursing workflow<br>These mechanisms therefore remain speculative.<br><br>6. Definition of probable CLABSI<br>The definition of the primary outcome requires clarification.<br>The term "probable CLABSI" is confusing because the definition requires microbiological confirmation together with clinical findings and prolonged antibiotic treatment. The surveillance definition used should be clearly identified, and the implications of incorporating treatment duration into the outcome definition should be discussed.<br><br>7. Antimicrobial pressure<br>The manuscript repeatedly refers to "antimicrobial pressure," whereas the reported outcome is antibiotic Days of Therapy (DOT). DOT is a measure of antimicrobial exposure rather than antimicrobial selective pressure. More precise terminology should be used throughout.<br><br>8. Sample size<br>The sample size calculation appears inconsistent with the primary outcome.<br>The calculation is based on a 20% composite outcome and an expected 80% relative reduction, whereas the primary endpoint is CLABSI incidence per 1,000 catheter-days analysed using Poisson regression.<br>The assumptions underlying the calculation require clarification.<br><br>9. Mortality<br>The mortality analysis is underpowered.<br>Only 14 deaths occurred, resulting in very wide confidence intervals. Mortality should therefore be presented as an exploratory outcome, and, if available, causes of death should be reported to allow readers to judge any relationship with bloodstream infection.<br><br>10. Discussion<br>The Discussion requires substantial revision.<br>It currently:<br>• overattributes the observed reduction to the intervention,<br>• provides limited critical appraisal,<br>• does not sufficiently discuss alternative explanations,<br>• largely ignores the high residual CLABSI rate,<br>• omits discussion of important contemporary prevention strategies.<br>Particularly, the Discussion should acknowledge evidence supporting:<br>• insertion and maintenance bundles,<br>• vascular access teams,<br>• catheter maintenance protocols,<br>• implementation science,<br>• cyanoacrylate tissue adhesive as an adjunct to catheter stabilization and sealing.<br>The manuscript should discuss whether CMLIS provides an incremental benefit once these established measures have been optimized rather than implying that it represents the primary solution to CLABSI prevention.

	Minor comments<br>Crude versus adjusted estimates<br>The manuscript combines crude incidence rates with adjusted effect estimates.<br>For example, the observed reduction from 16.42 to 9.05 per 1,000 catheter-days corresponds to an approximate 45% crude reduction, whereas the reported 50% reduction reflects the adjusted incidence rate ratio (aIRR = 0.50). These measures should be presented separately.<br><br>Terminology<br>Several terms would benefit from simplification, including:<br>• probable CLABSI<br>• antimicrobial pressure<br>• therapeutic antibiotic consumption<br><br>Microbiology<br>If the proposed mechanism involves reduced catheter hub contamination, reporting the microbiological spectrum of bloodstream infections would considerably strengthen the biological plausibility of the findings.<br><br>Overall recommendation<br>In its current form, I have substantial reservations regarding the scientific framing and interpretation of this study. The observational association between CMLIS implementation and reduced CLABSI is interesting, but the manuscript does not adequately demonstrate that the infusion system was the principal remaining contributor to the unit's infection burden, nor does it sufficiently position the intervention within established evidence-based CLABSI prevention strategies.<br>The Discussion, in particular, should begin by acknowledging that CMLIS is likely to be one component of a multifactorial infection prevention programme, rather than presenting it as the primary solution. Without this conceptual reframing and a substantially more balanced interpretation, I do not believe the manuscript supports the strength of its conclusions.<br><br>Final comment<br>I believe the manuscript currently starts at the end of the quality improvement process rather than at the beginning. The discussion should therefore be reframed to first address the broader causes of the high baseline CLABSI rate and to clarify where the closed infusion system fits within a comprehensive CLABSI prevention programme. In my view, advanced technologies such as closed infusion systems should be evaluated as adjunctive interventions rather than as primary solutions to an unresolved infection problem. Before introducing a novel device, the underlying causes of a persistently high CLABSI rate should be systematically investigated and established evidence-based prevention strategies optimized. Only then can the incremental value of a new technology be appropriately assessed.<br><br>The authors may find the following publication helpful in framing this discussion, as it explores the role of closed intravenous systems within the broader context of neonatal CLABSI prevention rather than as a standalone intervention:<br>van Rens MFPT, Hugill K, Francia ALV, et al. Closed intravenous systems for central vascular access: A difference maker for CLABSI

	rates in neonates? Journal of Vascular Access. 2023;24(6):1390–1397. doi:10.1177/11297298221085480.

**REVIEWER NAME**	Picaud, Jean-Charles
**REVIEWER AFFILIATION**	Hopital croix rousse, Neonatology
**REVIEWER CONFLICT OF INTEREST**	No Conflict of Interest
**AI DISCLOSURE**	No, I have not used AI
**DATE REVIEW RETURNED**	14-Jul-2026

**GENERAL COMMENTS**	General comment<br>The authors present the second paper evaluating the impact of a new closed multi-infusion system in very preterm neonates. They confirm a significant reduction in the CLABSI rate, together with a reduction in antibiotic exposure. Although this is a retrospective study, it contributes evidence suggesting that use of the new device could help improve the health of very preterm infants. The results should be compared more carefully with the only other available study evaluating the new device. Improvements to the manuscript are needed to enhance its quality.<br><br>Point-by-point comment<br>• Point 1, P3, L6, Abstract: Was the study registered (ClinicalTrials.gov or another registry)? If so, please provide the registration number.<br>• Point 2, P3, L8, Abstract: The Background section should include the fact that the first study to evaluate the new device showed a drastic reduction in CLABSI rates, and that the objective of the present study was to evaluate whether implementing the same device in another unit with different practices could confirm this impact.<br>• Point 3, P3, L10, Abstract: As multi-infusion is the main topic of the paper, the authors should use "multi-infusion" instead of "infusion." Single infusion systems do not require as many manipulations and are not associated with the same risk of bacteriological contamination.<br>• Point 4, P4, L8, What is already known: "Multi-infusion systems" rather than "infusion systems" (see Point 3).<br>• Point 5, P4, L10, What is already known: Two points should be presented in this section to clarify the Background: 1) data available in adults suggest that the CLABSI rate increases when a standard multilumen infusion system is used (Dezfulian 2003, DOI: 10.1097/01.CCM.0000084843.31852.01), and 2) the use of new closed multilumen infusion systems has been associated with a strong decrease in CLABSI rates in neonates. A possible wording: "The CLABSI rate increases when standard multi-infusion systems are used, but it has recently been suggested that a new closed multilumen system could help drastically reduce CLABSI rates in neonates."<br>• Point 6, P4, L17–18, What this study adds: The term "confirm" should be preferred to "suggest," as the current article confirms the results first reported on the use of this device in a neonatal unit.<br>• Point 7, P4, L19, What this study adds: The authors should present the reduction in antibiotic "exposure" rather than "consumption," because results are expressed as days of treatment and the objective is to show an impact on antibiotic exposure and its consequences on the microbiota. Furthermore, "exposure" is the term used by the authors in the title.

	• Point 8, P4, L24, How this study might affect research, practice, or policy: It should be highlighted that the main impact on practice is the reduction in the CLABSI rate (and consequently a reduction in antibiotic exposure), which has a major impact on the health of neonates, and also on parental stress. It should also have an impact on policy regarding the duration of multi-infusion system use, since it is currently recommended to change the system every 7 days, whereas the new device can be used for 21 days. This is the second study showing that, despite the catheter being left in place for longer, a reduction in CLABSI is observed. Although both studies are retrospective, they each show a major reduction in CLABSI rates in two different neonatal units — i.e., with different practices, particularly regarding hygiene rules and the procedures for administering intravenous infusions.<br>• Point 9, P4, L26, How this study might affect research, practice, or policy: "Multi-infusion" rather than "infusion" (see Point 3).<br>• Point 10, P5, L17–18, Introduction: This sentence should be corrected to reflect the source more accurately: in reference 4 (Klinger 2024), 20.4% of infants born at 24–28 weeks had LOS, ranging from 7.1% to 44.6%. The 20.6% reported by the authors of the current article is in agreement with a large Canadian survey (Zipursky et al. 2019, DOI: 10.1016/j.jpeds.2018.12.011), which reported a 21.6% LOS rate in infants <28 weeks. It should also be added that three out of every four cases of LOS are CLABSI (Zipursky et al. 2019), as this is the main topic of the submitted article.<br>• Point 11, P5, L35–36, Introduction: "Multi-infusion" rather than "infusion" (see Point 3).<br>• Point 12, P5, L35–36, Introduction: "Traditional infusion systems rely on manifolds and stopcocks...". Not only. Manifolds and stopcocks are the simplest devices, although they are used in a wide variety of combinations in neonatal units. There are also more complex multi-infusion systems (ICU, Octopus), but all of these are open multi-infusion systems, which is the main difference from the CMLIS evaluated in the current article. This is a crucial point, explaining the large differences in how the multi-infusion device is used.<br>• Point 13, P5, L42, Introduction: The authors should improve their explanation of the CMLIS evaluated, compared with previously available standard devices, including the fact that this CMLIS can be used for up to 21 days (ref 3).<br>• Point 14, P5, L47–48, Introduction: The beginning of the sentence should be corrected to highlight that there was only one previous evaluation, carried out in France — the only country where the CMLIS is currently available. It should read: "While the first published evaluation suggests..." The end of the sentence should specify the "knowledge gaps."<br>• Point 15, P5, L49–56, Introduction: The methodological limitations should be explained in more detail. Admittedly, only randomized controlled trials will be able to "establish a definitive impact." However, the authors proposed to "overcome methodological limitations" by improving the methodology of this retrospective before-and-after study. Indeed, they used an improved analytical approach (multivariate regression models followed by propensity score matching as a sensitivity analysis), but ultimately confirmed the results presented in reference 3. This should be discussed: are the differences in the magnitude of CLABSI rate reduction (-51% vs. -88%) related to differences in statistical methodology, in the populations studied, in the type of central venous line used, in the criteria used to define CLABSI, and/or to the high CLABSI rate

	(16.4/1000 catheter-days)?<br>• Point 16, P6, L11-16, Ethics: Please provide more information about parental consent.<br>• Point 17: P7, L3, Methods: Please describe more precisely: 1) the setting (number of nurses in the intervention NICU, open-bay or single-room unit, etc.), 2) the training process (who was trained, the time elapsed between the start of training and implementation of Multiline, etc.), and 3) the multilumen system used (or refer to reference 3 if strictly identical), as these factors may affect the results.<br>• Point 18: P7, L11, Methods: Definition of LOS and CLABSI. Since this is the main outcome measure, please clarify the term "probable." The following sentence refers to a French report (reference 11, to be corrected); it is likely that a widely used international definition was applied in that report. Please cite it directly or provide more information on the definition used. Did you define infections as "probable" (clinical sepsis without a positive blood culture) and "certain," and if so, using which criteria? Please provide more information so that your data can be compared with previously published results.<br>• Point 19: P7, L 23, Methods: Please clarify the definition of LOS (see comment above).<br>• Point 20: P7, L 23, Methods: Replace "pressure" with "exposure" (cf. title). Please clarify what information was collected: total antibiotic exposure during hospitalization? Only certain antibiotics? Only over a given period?<br>• Point 21: P7, L27, Methods: Does this refer to the total hospital stay (NICU, intermediate care unit, and standard care unit), or only the NICU stay?<br>• Point 22: P7, L29: "...bronchopulmonary dysplasia (BPD) at 36 weeks [12]..." refers to an outdated definition of BPD that is no longer in use. Please use the Jensen et al. definition (DOI: 10.1164/rccm.201812-2348OC) or explain why the older definition was used.<br>• Point 23: P7, L33-34, Methods: Necrotizing enterocolitis (NEC) greater than stage 2 (14)": the currently used reference is the one updated by Walsh and Kliegman (DOI: 10.1016/s0031-3955(16)34975-6)<br>• Point 24: P7, L40, Methods: The before-after design compares a historical cohort (2024) with a contemporary cohort (2025). The power calculation is therefore actually a post hoc calculation of available power, since sample sizes are fixed by the number of patients admitted each year rather than chosen to reach a target size. There is therefore a risk of temporal confounding (concurrent practice changes, seasonal effects, other interventions introduced in the same year) that is not captured by a power calculation. It would be more accurate to present this as an estimate of available power given the expected sample size, rather than as a required sample size in the sense of a prospective trial with controlled recruitment.<br>• Point 25: P7, L47, Methods: Please clarify what is meant by "composite outcome," since line 11 of the same page states that the main outcome is the "incidence of probable CLABSI."<br>• Point 26, P7, L51-52, Methods: Such a reduction in the CLABSI rate was previously reported (ref 3). If different criteria were used than in ref 3 — a "composite" criterion rather than the original one — can such a large effect really be expected? See comment above.<br>• Point 27, P10, L13, Results: Results are presented as mean ± SD or as median and IQR in both the text and the tables. For consistency throughout the manuscript, it would be preferable to present the data uniformly as median and IQR.

	• Point 28, P10, L25, Results: Table 1: Please correct the sentence, as there are no biological data in this table. The Methods section states that morbidity data (NEC grade ≥2, moderate or severe BPD, ROP, etc.) were collected, but these results are not presented in Table 1. Please also present LOS, duration of parenteral nutrition, and postnatal steroid exposure in both groups. This is needed to assess whether the study population is comparable to infant populations in other neonatal units (generalizability).<br>• Point 29, P10, L33, Results: Please present more details on the central venous line and the type of catheter used in infants born before or after implementation of the CMLIS, including catheter duration and the catheter utilization ratio (number of catheter-days divided by the number of patient-days). This is needed for a rigorous interpretation of the results.<br>• Point 30, P12, L12, Discussion: The CLABSI rate was high before implementation of the CMLIS — higher than the most recent values (12.4 [10.8–14] per 1000 catheter-days) published at the national level in France (CPIAS 2019). This should be discussed, as it reflects practices that could be improved and may explain why implementation of the CMLIS had a smaller effect than in the first published evaluation.<br>• Point 31, P12, L31-34, Discussion: The reference should be changed, as ref. 22 does not report "evidence supporting minimal handling protocols in neonatal intensive care," but merely an evaluation of replacing umbilical venous catheters with percutaneously inserted central catheters.<br>• Point 32, P12, L38, Discussion: "Infections" should be made more precise — replace with "CLABSI."<br>• Point 33, P12, L41, Discussion: “The reduction in infections may be attributed to ... and protecting vulnerable infants from environmental stress by moving the infusion hub outside the incubator.” This sentence should be revised, as "protection from environmental stress" has no direct impact on infection risk, but rather supports the infant's physiological stability by reducing the number of interventions and sleep interruptions. Other reasons why the CMLIS might help reduce the CLABSI rate should be presented: a closed-line system avoiding repeated connection/disconnection, tubing changes every 21 days instead of 7, etc. It should also be emphasized that this reduction in CLABSI rate was observed even though the duration of catheter use was longer than the 7 days usually recommended for changing the open multi-lumen infusion systems currently in use.<br>• Point 34, P13, L8-15, Discussion: The reduction in CLABSI rate and antibiotic exposure is unlikely to reduce length of hospital stay or mortality in a population exposed to so many other confounding factors; it is more likely to affect outcomes related to gut microbiota and dysbiosis, such as digestive tolerance, NEC, duration of parenteral nutrition, and postnatal growth.<br>• Point 35, P13, L18-22, Discussion (see point 15). The authors refined the analytical methodology, but ultimately confirmed the results already reported in reference 3. This should be discussed further. Are the differences in the magnitude of CLABSI rate reduction (-51% vs. -88%) related to differences in the statistical methods used, the study populations, the type of central venous line, the criteria used to define CLABSI, and/or the high baseline CLABSI rate (16.4/1000 catheter-days)?<br>• Point 36, P13, L27, Discussion: Additional limitations should be acknowledged, such as the study not being powered to detect an effect on neonatal morbidities, etc.<br>• Point 37: references should be checked and corrected.

REVIEWER NAME	Bryant, Kristina
REVIEWER AFFILIATION	
REVIEWER CONFLICT OF INTEREST	No Conflict of Interest
AI DISCLOSURE	No, I have not used AI
DATE REVIEW RETURNED	21-Jul-2026

GENERAL COMMENTS	Dacquino and colleagues describe the impact of a closed multi-lumen infusion system (CMLIS) on probable central-line associated bloodstream infections and antibiotic use in preterm neonates.<br><br>Many neonatal intensive care units continue to see CLABSIs in preterm infants despite reliable implementation of evidence-based bundles, making evaluation of new strategies and technologies important.<br><br>The authors refer several times to "very preterm infants." The World Health Organization defines "very preterm" as 28 to less than 32 weeks gestation. It would be good to clarify if this is the definition intended by the authors or they are using the term more generally.<br><br>In the Methods section, it would be beneficial to provide more details about the neonatal intensive care unit that conducted the study, including number of beds and annual admissions.<br><br>The title and abstract of the work reference CLABSI as an outcome. In the Outcomes section, the authors clarify that the primary aim of the study is to compare the incidence of "probable CLABSI" in infants managed with standard infusion sets or CMLIS. Is "probable CLABSI" part of national infection prevention surveillance definitions in France or was this a definition adopted for this study? Was the outcome assessed by the investigators or the hospital's infection prevention team?<br><br>The sample size calculation was based on a pre-intervention composite outcome rate. Could this composite outcome rate be defined in the paper?<br><br>The term "probable CLABSI" should be used throughout the paper, including in the results section and tables.<br><br>The definition of probable CLABSI included a single positive blood culture. The potential that some of the patients with probable CLABSI could have actually had contaminated blood cultures should be acknowledged as a limitation.<br><br>It would be beneficial to know the distribution of organisms associated with positive blood cultures.<br><br>In the Discussion section, the authors note that a temporal effect cannot be excluded based on the retrospective nature of study. Were there any other temporal trends that could have impacted infection rates, such as improved hand hygiene compliance or central line bundle reliability? If not, it would be good to explicitly state this.<br><br>A CLABSI rate of 16.42 per 1000 catheter days is relatively high.

	Even while acknowledging that the study was measuring probable CLABSI, this high baseline rate may limit generalizability to other NICUs.

VERSION 1 – AUTHOR RESPONSE

Reviewer #1
Mr. Roland van Rens, Hamad Medical Corporation

Overall assessment
This manuscript addresses an important clinical question and reports an association between implementation of a closed multi-lumen infusion system (CMLIS) and reduced CLABSI incidence in a single tertiary NICU. However, I have significant concerns regarding the underlying scientific rationale, interpretation of the findings, and discussion.
The central premise of the study appears to be that changing the infusion system is the primary strategy to reduce CLABSI. However, CLABSI is a multifactorial complication, and contemporary prevention relies on a comprehensive quality improvement programme including standardized insertion and maintenance bundles, vascular access expertise, catheter securement, hub disinfection, staff competency, audit and feedback, and implementation science. The manuscript does not describe whether these established strategies were already fully implemented or whether the underlying causes of the relatively high baseline CLABSI rate had been systematically investigated.
In my opinion, this represents the principal weakness of the manuscript. Before evaluating an advanced technological intervention, it is essential to understand the causes of the existing infection burden and demonstrate that the remaining infections are likely attributable to the aspect of care targeted by the intervention. Without this context, the study cannot determine whether the observed improvement reflects the effect of the infusion system itself or broader changes in clinical practice.
For this reason, I believe the Discussion requires substantial revision and should begin by acknowledging this limitation.

Reviewer Comment:
1. Scientific rationale
The fundamental scientific rationale requires reconsideration.
The baseline CLABSI incidence reported in this study is 16.4 per 1,000 catheter-days, decreasing to 9.05 per 1,000 catheter-days following implementation of the CMLIS. Although this represents a reduction, the post-intervention rate remains relatively high, indicating that the underlying problem has not been resolved.
In quality improvement, advanced technological interventions are generally introduced after established infection prevention strategies have been optimized and residual causes of failure have been systematically investigated. The manuscript does not describe whether such an evaluation was undertaken.
Specifically, there is no information regarding:
• insertion bundle compliance
• maintenance bundle compliance
• vascular access team involvement
• catheter care audits
• hub disinfection compliance
• dressing integrity
• catheter securement
• root-cause analyses
• failure mode analysis
• concurrent quality-improvement initiatives
Without demonstrating that excessive infusion system manipulation was the dominant remaining

contributor to CLABSI, it is difficult to conclude that the observed reduction is primarily attributable to the CMLIS.
I strongly recommend that the Discussion begins by acknowledging this limitation and positioning the intervention as one potential component of a comprehensive CLABSI prevention strategy rather than the principal solution.

Response: we completely agree with the core principle of Quality Improvement highlighted here: advanced technological devices should never be leveraged as a substitute for optimized, foundational infection prevention practices, nor should they be used to bypass a systematic investigation of care bundles. To address the Reviewer’s concerns regarding our baseline rates and the clinical context of the intervention, we have performed a thorough review of our database and restructured the manuscript accordingly:
Upon re-verifying our full cohort data, the overall crude unadjusted CLABSI incidence density was 15.38 per 1,000 catheter-days in the Control group and 8.27 per 1,000 catheter-days in the CMLIS group. While these overall numbers may appear elevated at first glance, they reflect a highly fragile population with a very high proportion of Extremely Low Birth Weight (ELBW) and Very Low Birth Weight (VLBW) infants requiring non-umbilical central venous catheters (CVCs). When stratifying the incidence rates by birth weight, our baseline rates are entirely comparable to established international benchmarks. Specifically, we compared our data with the national report from the French Néocat registry (Mission Müller, CPIAS. Surveillance des infections nosocomiales en réanimation néonatale. Rapport National), which focuses on non-umbilical CVCs in equivalent neonatal intensive care settings:
Birth Weight Category Control Group CLABSI Rate (Our Study) French Néocat Registry (Benchmark)
750 g or less 20.8 22.0
751 to 1,000 g 17.8 17.2
1,001 to 1,500 g 8.7 8.4

As shown in the table, our baseline infection rates reflect the inherent biological and clinical vulnerability of these neonates rather than a systematic failure of baseline hygiene. Within this optimized framework, the CMLIS was introduced to provide an incremental, targeted benefit against line manipulation-related risks.
We have significantly expanded the Methods section (page 7, lines 1-8) to clarify that a solid, structured framework of evidence-based infection control practices was already active and optimized in our unit before and during the study period. This includes standardized insertion and maintenance bundles, strict hub-disinfection protocols, and regular nursing audits. The CMLIS was not introduced to compensate for poor compliance, but rather as an additional layer of safety to minimize fluid path contamination during mandatory manifold manipulations.
We have restructured the Discussion (page17, lines 10-16) to transparently acknowledge that formal, quantified compliance audits for each specific bundle element (e.g., precise tracking of hub-disinfection seconds, formal failure mode analyses, or root-cause analyses) were not systematically recorded in a structured database for this study. We now explicitly present the CMLIS as an effective, complementary component of a multifaceted CLABSI prevention strategy.

2. Study design and interpretation
The Introduction appropriately critiques previous observational before-and-after studies for their susceptibility to temporal bias. However, the present investigation employs the same underlying historical cohort design, although strengthened by multivariable adjustment and propensity score matching.
These statistical methods improve adjustment for measured confounders but cannot eliminate residual confounding or secular trends. The manuscript should therefore avoid implying causal inference.
Response : While multivariable adjustment and Propensity Score Matching significantly enhance the

balance of observed baseline covariates, they cannot completely mitigate unmeasured residual confounding or underlying secular trends inherent to historical before-and-after designs.
We have thoroughly revised the manuscript to ensure that we do not imply or claim direct causal inference. Specifically, we have performed the following adjustments:
- We have systematically scanned the text (including the Title, Abstract, Objectives, and Results) to replace causal language (such as "impact" or "effect") with associative language (such as "association," "associated with," or "trend").
- The final paragraph of the Introduction (page 4, line 17) has been reworded to transparently state that our advanced statistical framework is an effort to mitigate, rather than eliminate, the limitations of historical cohorts.
- We have expanded the limitations section in the Discussion (Page 17, lines 4-9) to explicitly clarify that statistical matching cannot account for unmeasured confounders (e.g., secular shifts in nursing care patterns or temporal biases) and that our findings demonstrate a robust statistical association rather than definitive causality.

3. Study objective
The objective is clearly presented in the Abstract but is not explicitly stated at the end of the Introduction. A concise statement of the research question would improve the manuscript structure.

Response : We agree that explicitly stating the research question at the closing of the Introduction (page 5, lines 14-16) improves the clarity and flow of the manuscript. Accordingly, we have revised the final paragraph of the Introduction to incorporate a concise and explicit statement of both our primary research objective.

4. Background
The Introduction does not adequately position the intervention within current neonatal vascular access practice.
Evidence-based strategies including insertion and maintenance bundles, vascular access teams, standardized catheter care, catheter securement, hub disinfection, audit programmes, implementation science, and cyanoacrylate tissue adhesive should be discussed before introducing the CMLIS.
Response: We entirely agree with the Reviewer that the CMLIS should be contextualized as an evolution of, rather than a substitute for, established evidence-based vascular access practices. We appreciate this opportunity to clarify that our tertiary NICU routinely enforces these high-standard preventive strategies, including dedicated vascular access protocols, strict hub disinfection, routine audits, and the use of cyanoacrylate tissue adhesive for catheter securement. In accordance with the Reviewer's suggestion, we have substantially revised and expanded the Introduction (page 4, lines 21-25, and page 7 lines 1-8). We have now explicitly reviewed the roles of insertion/maintenance bundles, specialized teams, implementation science framework, audit programs, and recent innovations like cyanoacrylate adhesives. This addition allows us to properly position the CMLIS as a targeted structural intervention designed specifically to address the residual risks of intraluminal contamination, building upon an already optimized foundation of standard infection control.

5. Mechanism
The Discussion repeatedly proposes that reduced manipulation led to reduced contamination and fewer infections.
However, the study did not measure:
• manipulation frequency
• catheter accesses
• hub contamination
• incubator openings
• nursing workflow
These mechanisms therefore remain speculative.

Response : Because our study was retrospective, we did not prospectively track or quantify clinical behaviors (such as the exact number of line manipulations or incubator openings) or microbiological markers (such as hub colonization cultures). Therefore, proposing these factors as definitive causal mechanisms is indeed speculative.
We have thoroughly revised the Discussion section to address this limitation:
- We have toned down the language in the text, explicitly framing the role of reduced manipulation and incubator openings as biologically plausible hypotheses rather than demonstrated facts.
- We have added a dedicated paragraph in the limitations section of the Discussion (page 17, lines 10-16) to transparently state that manipulation frequency, catheter accesses, hub contamination, incubator openings, and nursing workflow changes were not measured, leaving the precise underlying mechanisms open to further prospective verification.

6. Definition of probable CLABSI
The definition of the primary outcome requires clarification.
The term "probable CLABSI" is confusing because the definition requires microbiological confirmation together with clinical findings and prolonged antibiotic treatment. The surveillance definition used should be clearly identified, and the implications of incorporating treatment duration into the outcome definition should be discussed.
Response : the term "probable" is epidemiologically inaccurate and potentially misleading, given that our definition strictly required microbiological confirmation alongside clinical signs.
We have revised the manuscript as follows:
- We have removed the term "probable CLABSI" and replaced it with " CLABSI." We have explicitly identified that our primary outcome aligns with the Center for Disease Control and Prevention / National Healthcare Safety Network (CDC/NHSN) surveillance framework, adapted with a clinical and therapeutic threshold to maximize specificity in a neonatal cohort (Page 8 lines 3-8).
- We have added a dedicated paragraph in the limitations section (Page 17, lines 20-24) discussing the implications of including a 5-day antibiotic treatment rule. We explain that while this choice significantly reduces the risk of misclassifying transient skin contaminants (such as Coagulase-Negative Staphylococci) as true infections, it represents a conservative approach that could theoretically underestimate the incidence in patients facing early mortality or altered treatment courses.

7. Antimicrobial pressure
The manuscript repeatedly refers to "antimicrobial pressure," whereas the reported outcome is antibiotic Days of Therapy (DOT).
DOT is a measure of antimicrobial exposure rather than antimicrobial selective pressure. More precise terminology should be used throughout.
Response : We completely agree that Days of Therapy (DOT) strictly quantifies antimicrobial exposure and consumption, whereas "antimicrobial pressure" implies the ecological consequence of selection for resistance. Using these terms interchangeably was imprecise.
We have thoroughly revised the manuscript to ensure accurate terminology:
- We have replaced "antimicrobial pressure" with "antimicrobial exposure" or "antibiotic consumption" throughout the Abstract, Methods, and Results sections (including text, tables, and outcomes definitions).
- In the Discussion (Page 16, lines 10-12), we have rephrased the sentence to clarify that the observed reduction in vancomycin exposure is clinically valuable because it represents a strategy that could potentially limit the subsequent selective pressure for multidrug-resistant organisms in the NICU.

8. Sample size
The sample size calculation appears inconsistent with the primary outcome.

The calculation is based on a 20% composite outcome and an expected 80% relative reduction, whereas the primary endpoint is CLABSI incidence per 1,000 catheter-days analysed using Poisson regression.
The assumptions underlying the calculation require clarification.

Response : We apologize for the confusion. The mention of a "20% composite outcome and an 80% relative reduction" was a leftover from the earliest phase of the study protocol, where the primary endpoint was originally conceptualized as a composite outcome, representing the proportion of patients experiencing CLABSI or death. However, to ensure the highest methodological and epidemiological rigor, we subsequently shifted our primary outcome from a composite outcome to CLABSI incidence density per 1,000 catheter-days, analyzed under a Poisson framework. We omitted to update the sample size text accordingly in the previous draft, but we have now completely rewritten the statistical power section in the Methods (page 9, lines 2-12) using the correct assumptions for a Poisson rate framework based on our actual, fixed cohort data. Under this updated framework, the post hoc power estimation assumes a primary endpoint of CLABSI episodes per 1,000 catheter-days. Based on a total exposure of 3,252 catheter-days, a baseline historical control rate of 15.38 per 1,000 catheter-days, and a two-sided alpha of 0.05, this available sample size provided greater than 80% power to detect an incidence rate ratio less than or equal to 0.50 in the CMLIS group. This update resolves the inconsistency and aligns the power description perfectly with the final regression models presented in the Results.

9. Mortality
The mortality analysis is underpowered.
Only 14 deaths occurred, resulting in very wide confidence intervals.
Mortality should therefore be presented as an exploratory outcome, and, if available, causes of death should be reported to allow readers to judge any relationship with bloodstream infection.

Response : We entirely agree with the Reviewer's rigorous assessment regarding the statistical power of the mortality analysis. Given that only 14 death events occurred across the entire cohort, any definitive conclusions regarding mortality are inherently limited by wide confidence intervals and a clear lack of statistical power. In accordance with the Reviewer's excellent recommendation, we have revised the manuscript to explicitly reframe the mortality analysis as a purely exploratory outcome. This distinction has been clarified in the Statistical Analysis subsection within the Methods (page 10, lines 18-22), as well as in the narrative of the Results section (page 13, lines 13-20), ensuring that readers do not misinterpret these findings as a primary or fully powered endpoint.
Furthermore, to allow readers to critically judge any potential relationship between CLABSI and mortality, we have now incorporated a detailed breakdown of the causes of death for these 14 neonates in the Results section (page 13, lines 13-20). Specifically, 5 cases were secondary to severe intraventricular hemorrhage (IVH), 4 to persistent pulmonary hypertension of the newborn (PPHN) with obstructive shock, and 4 to necrotizing enterocolitis (NEC), while specific cause-of-death data were unavailable for 1 patient. Crucially, no deaths across the entire cohort were clinically attributed to septic shock or bloodstream infections. This qualitative addition provides the necessary clinical context, demonstrating that the fatal outcomes were entirely driven by severe baseline neonatal comorbidities rather than being directly attributable to catheter-related infectious failures. In the revised manuscript, these modifications have been integrated into the final paragraph of the Results section (page 13, lines 13-20) and are transparently addressed as a methodological limitation in the Discussion (page 17, lines 17-19).

10. Discussion
The Discussion requires substantial revision.
It currently:
• overattributes the observed reduction to the intervention,

• provides limited critical appraisal,
• does not sufficiently discuss alternative explanations,
• largely ignores the high residual CLABSI rate,
• omits discussion of important contemporary prevention strategies.
Particularly, the Discussion should acknowledge evidence supporting:
• insertion and maintenance bundles,
• vascular access teams,
• catheter maintenance protocols,
• implementation science,
• cyanoacrylate tissue adhesive as an adjunct to catheter stabilization and sealing.
The manuscript should discuss whether CMLIS provides an incremental benefit once these established measures have been optimized rather than implying that it represents the primary solution to CLABSI prevention.

Responce : We completely agree that framing any single device as a standalone solution to CLABSI is epidemiologically inaccurate and misrepresents the multifaceted nature of modern infection control. In accordance with the Reviewer's recommendations, we have fundamentally restructured the Discussion (page 15-17) to present the Closed Multi-line Infusion System (CMLIS) strictly as an adjunctive, incremental safeguard rather than a primary solution.
The revised section now shows our baseline and residual infection rates, framing them within the context of a highly fragile patient case-mix. To demonstrate that the CMLIS was introduced into an already optimized environment rather than to compensate for baseline flaws, we have detailed our quality-improvement ecosystem. This infrastructure explicitly includes standardized insertion and maintenance bundles, a dedicated vascular access team, strict catheter maintenance protocols, and the concurrent use of cyanoacrylate tissue adhesive for catheter stabilization and microbial site sealing. Furthermore, we have expanded our critical appraisal by introducing alternative explanations for the observed reduction, including unmeasured temporal trends and the potential behavioral impact of the pre-rollout training phase from an implementation science perspective. We have also expanded our limitations subsection to transparently acknowledge the lack of granular, prospective compliance data on day-to-day line management. We believe these revisions successfully reframe the CMLIS as a targeted tool designed to yield an incremental benefit only after foundational evidence-based measures have been systematically optimized.

Minor comments
Crude versus adjusted estimates
The manuscript combines crude incidence rates with adjusted effect estimates.
For example, the observed reduction from 16.42 to 9.05 per 1,000 catheter-days corresponds to an approximate 45% crude reduction, whereas the reported 50% reduction reflects the adjusted incidence rate ratio (aIRR = 0.50). These measures should be presented separately.

Response : We have thoroughly revised both the Abstract (page 1) and the main Results text (Page 12-13) to separate these measures explicitly.

Terminology
Several terms would benefit from simplification, including:
• probable CLABSI
• antimicrobial pressure
• therapeutic antibiotic consumption
Response : We have systematically revised the text to replace or simplify these phrases.

Microbiology
If the proposed mechanism involves reduced catheter hub contamination, reporting the

microbiological spectrum of bloodstream infections would considerably strengthen the biological plausibility of the findings.
We completely agree that detailing the microbiological spectrum considerably strengthens the biological plausibility of our proposed mechanism. In accordance with your suggestion, we have now included the microbiological isolates in the Results section (Page 12, line 23). Specifically, we report that Coagulase-Negative Staphylococci were the predominant pathogens across both periods, which aligns with typical neonatal CLABSI etiology. Since CoNS are the primary pathogens in these device-associated infections, their reduction directly supports the hypothesis of decreased catheter hub contamination. Furthermore, this specific microbiological profile perfectly contextualizes the observed 41% decrease in vancomycin days of therapy, as vancomycin represents our cornerstone therapy for CoNS. We believe this addition significantly clarifies the antimicrobial stewardship benefits observed in our cohort

Overall recommendation
In its current form, I have substantial reservations regarding the scientific framing and interpretation of this study. The observational association between CMLIS implementation and reduced CLABSI is interesting, but the manuscript does not adequately demonstrate that the infusion system was the principal remaining contributor to the unit's infection burden, nor does it sufficiently position the intervention within established evidence-based CLABSI prevention strategies.
The Discussion, in particular, should begin by acknowledging that CMLIS is likely to be one component of a multifactorial infection prevention programme, rather than presenting it as the primary solution. Without this conceptual reframing and a substantially more balanced interpretation, I do not believe the manuscript supports the strength of its conclusions.

Final comment
I believe the manuscript currently starts at the end of the quality improvement process rather than at the beginning. The discussion should therefore be reframed to first address the broader causes of the high baseline CLABSI rate and to clarify where the closed infusion system fits within a comprehensive CLABSI prevention programme. In my view, advanced technologies such as closed infusion systems should be evaluated as adjunctive interventions rather than as primary solutions to an unresolved infection problem. Before introducing a novel device, the underlying causes of a persistently high CLABSI rate should be systematically investigated and established evidence-based prevention strategies optimized. Only then can the incremental value of a new technology be appropriately assessed.

The authors may find the following publication helpful in framing this discussion, as it explores the role of closed intravenous systems within the broader context of neonatal CLABSI prevention rather than as a standalone intervention:
van Rens MFPT, Hugill K, Francia ALV, et al. Closed intravenous systems for central vascular access: A difference maker for CLABSI rates in neonates? Journal of Vascular Access. 2023;24(6):1390–1397. doi:10.1177/11297298221085480.

Response : We completely agree that exploring closed intravenous systems within the broader context of neonatal CLABSI prevention, rather than as a standalone intervention, provides a much stronger and more accurate perspective.
We have used the valuable insights from van Rens et al. as a foundational reference to restructure and frame our Discussion section. Accordingly, the manuscript has been revised to reflect this broader context, and the suggested article has now been added to our reference list (Reference 24, Page 21, line 18)

Reviewer #2
Dr. Jean-Charles Picaud, Hopital croix rousse, Universite Lyon 1 Faculte de Medecine et de Maieutique Lyon-Sud Charles Merieux
Comments to the Author

General comment
The authors present the second paper evaluating the impact of a new closed multi-infusion system in very preterm neonates. They confirm a significant reduction in the CLABSI rate, together with a reduction in antibiotic exposure. Although this is a retrospective study, it contributes evidence suggesting that use of the new device could help improve the health of very preterm infants. The results should be compared more carefully with the only other available study evaluating the new device. Improvements to the manuscript are needed to enhance its quality.

• Point 1, P3, L6, Abstract: Was the study registered (ClinicalTrials.gov or another registry)? If so, please provide the registration number.
Response : This study was a retrospective , non-interventional study based on routinely collected clinical data. Consequently, prospective registration in ClinicalTrials.gouv or another trial registry was not required.

• Point 2, P3, L8, Abstract: The Background section should include the fact that the first study to evaluate the new device showed a drastic reduction in CLABSI rates, and that the objective of the present study was to evaluate whether implementing the same device in another unit with different practices could confirm this impact.
Response : Framing our study as an independent replication and external validation of the pioneering data adds significant scientific value to the manuscript, highlighting the reproducibility of the technology across different intensive care settings with distinct baseline clinical practices.
We have completely revised the Background and Objective sections of the Abstract (page 2).

• Point 3, P3, L10, Abstract: As multi-infusion is the main topic of the paper, the authors should use "multi-infusion" instead of "infusion." Single infusion systems do not require as many manipulations and are not associated with the same risk of bacteriological contamination.
Response : In accordance with the Reviewer's recommendation, we have updated the terminology throughout the paper.

• Point 4, P4, L8, What is already known: "Multi-infusion systems" rather than "infusion systems" (see Point 3).

Response : In accordance with the Reviewer's recommendation, we have updated the terminology throughout the paper.

• Point 5, P4, L10, What is already known: Two points should be presented in this section to clarify the Background: 1) data available in adults suggest that the CLABSI rate increases when a standard multilumen infusion system is used (Dezfulian 2003, DOI: 10.1097/01.CCM.0000084843.31852.01), and 2) the use of new closed multilumen infusion systems has been associated with a strong decrease in CLABSI rates in neonates. A possible wording: "The CLABSI rate increases when standard multi-infusion systems are used, but it has recently been suggested that a new closed multilumen system could help drastically reduce CLABSI rates in neonates."
Response : In accordance with the Reviewer's recommendation, we have completely revised the "What is already known on this topic" section within the abstract summary box (page 3, lines 2-6) to incorporate both points and include the conceptual references to adult data (Dezfulian et al., 2003) and pioneering neonatal data (Picaud et al.). The full citations have also been integrated into the study's main bibliography.

• Point 6, P4, L17–18, What this study adds: The term "confirm" should be preferred to "suggest," as the current article confirms the results first reported on the use of this device in a neonatal unit.
Response : We have updated the "What this study adds" section in the abstract summary box (page 3, lines 9-14) to state that our findings corroborate that the implementation of a closed multi-lumen infusion system is associated with a decrease in CLABSI rates and a reduction in antibiotic exposure.

• Point 7, P4, L19, What this study adds: The authors should present the reduction in antibiotic "exposure" rather than "consumption," because results are expressed as days of treatment and the objective is to show an impact on antibiotic exposure and its consequences on the microbiota. Furthermore, "exposure" is the term used by the authors in the title.
Response : In accordance with the Reviewer's recommendation, we have updated the "What this study adds" section in the abstract summary box (page 3, line 11) to replace "antibiotic consumption" with "antibiotic exposure". We have also verified that this terminology is consistently applied throughout the entire manuscript text.

• Point 8, P4, L24, How this study might affect research, practice, or policy: It should be highlighted that the main impact on practice is the reduction in the CLABSI rate (and consequently a reduction in antibiotic exposure), which has a major impact on the health of neonates, and also on parental stress. It should also have an impact on policy regarding the duration of multi-infusion system use, since it is currently recommended to change the system every 7 days, whereas the new device can be used for 21 days. This is the second study showing that, despite the catheter being left in place for longer, a reduction in CLABSI is observed. Although both studies are retrospective, they each show a major reduction in CLABSI rates in two different neonatal units — i.e., with different practices, particularly regarding hygiene rules and the procedures for administering intravenous infusions.
Response : We have completely restructured the "How this study might affect research, practice, or policy" section in the abstract summary box (page 3, lines 17-23).

• Point 9, P4, L26, How this study might affect research, practice, or policy: "Multi-infusion" rather than "infusion" (see Point 3).
Response : In accordance with the Reviewer's recommendation, we have updated the terminology throughout the paper.

• Point 10, P5, L17–18, Introduction: This sentence should be corrected to reflect the source more accurately: in reference 4 (Klinger 2024), 20.4% of infants born at 24–28 weeks had LOS, ranging from 7.1% to 44.6%. The 20.6% reported by the authors of the current article is in agreement with a large Canadian survey (Zipursky et al. 2019, DOI: 10.1016/j.jpeds.2018.12.011), which reported a

21.6% LOS rate in infants <28 weeks. It should also be added that three out of every four cases of LOS are CLABSI (Zipursky et al. 2019), as this is the main topic of the submitted article.
Response : We have thoroughly revised the text to accurately disentangle the epidemiological benchmarks from Klinger et al. (2024) and Zipursky et al. (2019) (page 4, lines 7-9). Furthermore, we have integrated the crucial epidemiological detail that three out of four late-onset sepsis (LOS) cases are central line–associated bloodstream infections (CLABSIs) in this extremely vulnerable population, which provides a significantly stronger clinical justification for our study. The references have been updated and renumbered accordingly (page 19).

• Point 11, P5, L35–36, Introduction: "Multi-infusion" rather than "infusion" (see Point 3).
Response : In accordance with the Reviewer's recommendation, we have updated the terminology throughout the paper.

• Point 12, P5, L35–36, Introduction: "Traditional infusion systems rely on manifolds and stopcocks...". Not only. Manifolds and stopcocks are the simplest devices, although they are used in a wide variety of combinations in neonatal units. There are also more complex multi-infusion systems (ICU, Octopus), but all of these are open multi-infusion systems, which is the main difference from the CMLIS evaluated in the current article. This is a crucial point, explaining the large differences in how the multi-infusion device is used.
Response : The defining vulnerability shared by all traditional configurations,whether simple or highly complex,is their fundamentally open architecture. In accordance with the reviewers guidance, we have entirely rephrased this section to explicitly highlight the spectrum of traditional devices (from simple manifolds to complex multi-lumen extension lines) and to underscore that their "open" nature is the primary differentiator from the CMLIS. This correction significantly clarifies the pathophysiological and practical rationale behind the new device (page 5, lines 3-4).

• Point 13, P5, L42, Introduction: The authors should improve their explanation of the CMLIS evaluated, compared with previously available standard devices, including the fact that this CMLIS can be used for up to 21 days (ref 3).
Response : We have amended this sentence to provide a clearer contrast between the evaluated CMLIS and traditional standard configurations. Specifically, we have explicitly highlighted the pre-assembled closed design and incorporated the crucial technical specification regarding its extended lifespan of up to 21 days (page 5, line 10).

• Point 14, P5, L47–48, Introduction: The beginning of the sentence should be corrected to highlight that there was only one previous evaluation, carried out in France — the only country where the CMLIS is currently available. It should read: "While the first published evaluation suggests..." The end of the sentence should specify the "knowledge gaps."
Response : As recommended, we have thoroughly revised this sentence to clarify that the existing evidence is limited to a single study from Lyon, France. Furthermore, we have explicitly defined the "knowledge gaps" by stating that it remains to be determined whether these findings are reproducible in independent neonatal units operating under different baseline clinical practices and hygiene protocols (page 5, lines 13-14).

• Point 15, P5, L49–56, Introduction: The methodological limitations should be explained in more detail. Admittedly, only randomized controlled trials will be able to "establish a definitive impact." However, the authors proposed to "overcome methodological limitations" by improving the methodology of this retrospective before-and-after study. Indeed, they used an improved analytical approach (multivariate regression models followed by propensity score matching as a sensitivity analysis), but ultimately confirmed the results presented in reference 3. This should be discussed: are the differences in the magnitude of CLABSI rate reduction (-51% vs. -88%) related to differences in statistical methodology, in the populations studied, in the type of central venous line used, in the

criteria used to define CLABSI, and/or to the high CLABSI rate (16.4/1000 catheter-days)?
Response : While the initial prompt for this comment stemmed from the Introduction (page 5), we believe that a thorough, nuanced analysis of these divergent effect sizes (-46.2% unadjusted reduction in our cohort vs. -88% in the Lyon study) is best suited for the Discussion section (page 16) to avoid over-activating the Introduction.
As suggested, we have added a dedicated paragraph in the Discussion (page 16, lines 1-5) to explicitly address how our advanced statistical approach (multivariable adjustments and propensity score matching) inherently yields more conservative and epidemiologically realistic effect sizes. We have also highlighted that institutional variations in baseline infection prevention protocols could account for these differences.

• Point 16, P6, L11-16, Ethics: Please provide more information about parental consent.
Response : As this investigation was a retrospective study utilizing routinely collected, registry-based clinical data, the need for a specific, study-dedicated consent was waived by the Institutional Review Board. However, our institution enforces a strict, proactive consent protocol: upon NICU admission, all parents or legal guardians receive detailed written documentation explaining that de-identified clinical and surveillance data may be used for retrospective research and quality improvement purposes. Written informed consent for this institutional framework is systematically signed by the parents/guardians at admission, which includes a formal opt-out mechanism (the right to object to data utilization). We have explicitly detailed this framework in the revised text, ensuring full alignment with current European data protection regulations (GDPR) and the highest ethical standards for retrospective research.

• Point 17: P7, L3, Methods: Please describe more precisely: 1) the setting (number of nurses in the intervention NICU, open-bay or single-room unit, etc.), 2) the training process (who was trained, the time elapsed between the start of training and implementation of Multiline, etc.), and 3) the multilumen system used (or refer to reference 3 if strictly identical), as these factors may affect the results.
Response : In accordance with the Reviewer's recommendation, we have thoroughly revised the Methods section to incorporate these precise structural and operational characteristics:
- We have specified that our tertiary NICU is configured mostly with single-patient rooms (rather than an open-bay layout). We have also detailed the staffing matrix, clarifying that the unit maintains a strict 1:2 nurse-to-patient ratio, which is further supported by one support nurse for every 14 beds to manage clinical workflows (page 7, lines 15-19).
- We have expanded the description of our implementation protocol. A comprehensive, multi-disciplinary training programme was initiated 3 months prior to the clinical deployment of the system. This educational campaign was mandatory for all personnel accessing the lines, including attending physicians, pediatric residents, staff nurses, and nursing students (page 7, line 10).
- We have explicitly named the device used in our study, clarifying that we implemented the Edelweiss system, which is structurally and functionally identical to the CMLIS evaluated in the pioneering Lyon study (page 7, lines 12-14).

• Point 18: P7, L11, Methods: Definition of LOS and CLABSI. Since this is the main outcome measure, please clarify the term "probable." The following sentence refers to a French report (reference 11, to be corrected); it is likely that a widely used international definition was applied in that report. Please cite it directly or provide more information on the definition used. Did you define infections as "probable" (clinical sepsis without a positive blood culture) and "certain," and if so, using which criteria? Please provide more information so that your data can be compared with previously published results.
Response : We have revised the manuscript as follows:
- We have removed the term "probable CLABSI" and replaced it with " CLABSI." We have explicitly identified that our primary outcome aligns with the Center for Disease Control and Prevention / National Healthcare Safety Network (CDC/NHSN) surveillance framework, adapted with a clinical and

therapeutic threshold to maximize specificity in a neonatal cohort (Page 8, lines 3-8).
- We have added a dedicated paragraph in the limitations section (Page 17, lines 20-24) discussing the implications of including a 5-day antibiotic treatment rule. We explain that while this choice significantly reduces the risk of misclassifying transient skin contaminants (such as Coagulase-Negative Staphylococci) as true infections, it represents a conservative approach that could theoretically underestimate the incidence in patients facing early mortality or altered treatment courses.

• Point 19: P7, L 23, Methods: Please clarify the definition of LOS (see comment above).
Response : In accordance with the Reviewer's request, we have updated the Outcomes section (page 8, lines 13-15) within the Methods to explicitly define the criteria used for LOS:
- We specify that LOS defines any systemic infectious episode with an onset strictly after 72 hours of life.
- We clarify that an episode required the objective documentation of new-onset clinical signs of systemic instability paired with laboratory biomarker acceleration.
- In alignment with our primary outcome framework, we applied the same 5-day minimum systemic antibiotic therapy threshold to ensure that transient, self-limiting clinical fluctuations were not misclassified as true sepsis.
We explicitly state in the revised text that this definition encompasses both culture-confirmed episodes (including the subset of CLABSIs) and culture-negative clinical sepsis, providing a transparent framework for future comparative analyses.

• Point 20: P7, L 23, Methods: Replace "pressure" with "exposure" (cf. title). Please clarify what information was collected: total antibiotic exposure during hospitalization? Only certain antibiotics? Only over a given period?
Response : In accordance with the Reviewer's request for clarification, we have thoroughly updated the Outcomes section within the Methods to specify the exact boundaries of our antimicrobial data collection:
- We recorded all systemic antibiotics (intravenous and oral) administered to the study cohort. No therapeutic classes or specific drugs were omitted.
- The collection spanned the entire duration of the neonates' hospitalization within our tertiary NICU untill 36 weeks of GA.
- As already established in our protocol, we excluded only the empirical treatments initiated within the first 48 hours of life to prevent baseline early-onset sepsis management patterns from confounding the evaluation of the intervention.
We have integrated these details into the revised manuscript text, explicitly stating that the data represent cumulative daily exposure tracked via the Days of Therapy (DOT) methodology (page 8, lines 16-18).

• Point 21: P7, L27, Methods: Does this refer to the total hospital stay (NICU, intermediate care unit, and standard care unit), or only the NICU stay?
Response : We confirm that all data collection, length of stay metrics, catheter exposure days, and clinical outcomes refer strictly to the Neonatal Intensive Care Unit (NICU) stay, excluding any subsequent transfers to intermediate care units, step-down units, or standard wards.
In accordance with the Reviewer's suggestion, we have updated the Methods section to explicitly state that length of stay and associated metrics represent exclusively the duration of admission within our tertiary NICU, ensuring absolute clarity for comparative evaluations (page 8, line 12).

• Point 22: P7, L29: "...bronchopulmonary dysplasia (BPD) at 36 weeks [12]..." refers to an outdated definition of BPD that is no longer in use. Please use the Jensen et al. definition (DOI: 10.1164/rccm.201812-2348OC) or explain why the older definition was used.
Response : In accordance with the Reviewer's guidance, we have thoroughly revised the Methods

section (Page 8, line 20) to explicitly define the classic criteria used and provide the clinical rationale for maintaining this definition, while formally acknowledging the benchmark framework of Jensen et al. (2019).

• Point 23: P7, L33-34, Methods: Necrotizing enterocolitis (NEC) greater than stage 2 (14)": the currently used reference is the one updated by Walsh and Kliegman (DOI: 10.1016/s0031-3955(16)34975-6)
Response: In accordance with the Reviewer's correction, we have updated the Methods section (page 8, line 21) to explicitly cite the Walsh and Kliegman reference when defining proven NEC (stage > 2). The study bibliography has been amended accordingly, replacing the legacy reference.

• Point 24: P7, L40, Methods: The before-after design compares a historical cohort (2024) with a contemporary cohort (2025). The power calculation is therefore actually a post hoc calculation of available power, since sample sizes are fixed by the number of patients admitted each year rather than chosen to reach a target size. There is therefore a risk of temporal confounding (concurrent practice changes, seasonal effects, other interventions introduced in the same year) that is not captured by a power calculation. It would be more accurate to present this as an estimate of available power given the expected sample size, rather than as a required sample size in the sense of a prospective trial with controlled recruitment.
Response : We agree that in a retrospective before-and-after design, the sample size is structurally fixed by institutional admission rates, making any power assessment an evaluation of available statistical power rather than a prospective recruitment target. Furthermore, we acknowledge that a power calculation cannot capture or mitigate the risks of temporal confounding or secular trends inherent to comparing historical (2024) and contemporary (2025) cohorts.
In accordance with your guidance, we have entirely rewritten this section in the Methods to transparently reframe this as a post hoc estimation of available power within a convenience sample (page 9, lines 2-12). We have also explicitly added a statement acknowledging the limitation regarding unmeasured temporal confounding (page 17, lines 4-9).

• Point 25: P7, L47, Methods: Please clarify what is meant by "composite outcome," since line 11 of the same page states that the main outcome is the "incidence of probable CLABSI."
Response : The term "composite outcome" was a legacy error remaining from an earlier draft of the statistical protocol and does not reflect our actual analysis.
Our investigation features a single, clear primary outcome, which is the CLABSI incidence density (expressed per 1,000 catheter-days), with no composite variables utilized.
In accordance with the Reviewer’s note, we have corrected this sentence in the Sample size calculation section (page 9, lines 2-12) to explicitly replace "composite outcome" with "primary outcome", ensuring total alignment and consistency with the rest of the manuscript.

• Point 26, P7, L51-52, Methods: Such a reduction in the CLABSI rate was previously reported (ref 3). If different criteria were used than in ref 3 — a "composite" criterion rather than the original one — can such a large effect really be expected? See comment above.
Response : We completely understand the Reviewer’s concern and welcome this opportunity to provide absolute reassurance. As clarified in our response to Point 25, the inclusion of the phrase "composite outcome" in the statistical section was a pure typographical error from an earlier draft, which has now been completely removed. We explicitly confirm that no composite criteria were used in our study. Our primary outcome is strictly and exclusively defined as the of microbiologically confirmed CLABSI incidence density expressed per 1,000 catheter-days.

• Point 27, P10, L13, Results: Results are presented as mean ± SD or as median and IQR in both the text and the tables. For consistency throughout the manuscript, it would be preferable to present the

data uniformly as median and IQR.
Response : Given the inherently skewed distribution of key neonatal clinical characteristics (such as birth weight, gestational age, and duration of ventilation or hospitalization), presenting all continuous data uniformly as median and interquartile range (IQR) is mathematically more appropriate and significantly enhances the readability of the manuscript. In accordance with the Reviewer's guidance, we have thoroughly audited and updated the entire manuscript.

• Point 28, P10, L25, Results: Table 1: Please correct the sentence, as there are no biological data in this table. The Methods section states that morbidity data (NEC grade ≥2, moderate or severe BPD, ROP, etc.) were collected, but these results are not presented in Table 1. Please also present LOS, duration of parenteral nutrition, and postnatal steroid exposure in both groups. This is needed to assess whether the study population is comparable to infant populations in other neonatal units (generalizability).
Response : We have addressed each of your points as follows:
- We have corrected the sentence in the Results section, removing the word "biological" and replacing it with "demographic and clinical characteristics." (page 12, line 6).
- Morbidity Data, LOS, and PN Duration: We have now explicitly integrated all collected neonatal morbidities (NEC ROP IVH and BPD), late-onset sepsis (LOS) rates, and the duration of parenteral nutrition (PN) directly into Table 1 (page 23).
- Postnatal Steroid Exposure: Regarding postnatal corticosteroid exposure, we sincerely regret that due to the retrospective nature of our registry-based data collection, this specific variable was not systematically tracked or uniformly recorded in the clinical charts for this cohort, and is therefore unfortunately unavailable.
However, to preserve the structural generalizability requested, Table 1 (page 23) provides several highly robust indicators of baseline clinical severity and respiratory vulnerability. These include gestational age, birth weight, SNAPPE-II scores, the proportion of infants requiring prolonged invasive ventilation (>48h), and the overall incidence of bronchopulmonary dysplasia (BPD). These comprehensive markers clearly demonstrate that the control and CMLIS groups were highly comparable at baseline and closely match the profiles of extremely preterm infants managed in similar tertiary NICUs.

• Point 29, P10, L33, Results: Please present more details on the central venous line and the type of catheter used in infants born before or after implementation of the CMLIS, including catheter duration and the catheter utilization ratio (number of catheter-days divided by the number of patient-days). This is needed for a rigorous interpretation of the results.
Response: Following the reviewers recommendation, we have expanded the manuscript to include these specific details. We have added the information regarding the central venous lines to the Patients and Settings section (page 7, lines 12-13), specifying that across both periods, infants were managed using 2Fr polyurethane epicutaneo-caval catheters.
Furthermore, we have updated Table 1 (page 23) to include the catheter duration and the Catheter Utilization Ratio (CUR). Regarding catheter duration, there was no statistically significant difference between the two cohorts: the median catheter days were 12 [IQR: 8, 21] in the Control group compared to 11 [IQR: 7, 28] in the CMLIS group (p=0.53).
Importantly, the CUR was also comparable between the two groups (median 0.33 [IQR: 0.19, 0.60] for the Control group vs. median 0.28 [IQR: 0.18, 0.63] for the CMLIS group, p=0.80). This lack of significant difference in the CUR demonstrates that overall catheter exposure was essentially identical between the two groups, thereby eliminating exposure bias as a confounding factor in our infection rate analysis.

• Point 30, P12, L12, Discussion: The CLABSI rate was high before implementation of the CMLIS — higher than the most recent values (12.4 [10.8–14] per 1000 catheter-days) published at the national level in France (CPIAS 2019). This should be discussed, as it reflects practices that could be

improved and may explain why implementation of the CMLIS had a smaller effect than in the first published evaluation.
Response: We thank the reviewer for this crucial observation, which allows us to clarify a fundamental epidemiological aspect of our cohort.
While we agree that our overall, unstratified baseline CLABSI incidence density (15.38 per 1,000 catheter-days) appears higher than the raw national average published by CPIAS 2019, this discrepancy is entirely driven by a case-mix bias (i.e., a higher concentration of extremely low birth weight infants in our tertiary center compared to the mixed national sample).
When shifting from an unstratified rate to a weight-stratified analysis, our baseline data are entirely comparable to, and consistent with, the official French national registry data (Néocat registry) for non-umbilical central venous catheters. Specifically:
• For newborns ≤750 g: our rate was 20.8 vs. 22.0 per 1,000 catheter-days in Néocat.
• For newborns 751–1000 g: our rate was 17.8 vs. 17.2 per 1,000 catheter-days in Néocat.
• For newborns 1001–1500 g: our rate was 8.7 vs. 8.4 per 1,000 catheter-days in Néocat.
This demonstrates that our baseline infection rates reflected the high clinical severity and extreme vulnerability of our specific population rather than suboptimal hygiene or insertion practices. In fact, our center had already implemented an optimized, multi-component prevention program (including dedicated vascular access teams and cyanoacrylate tissue adhesive).
We have added a dedicated paragraph in the Discussion section (page 15, lines 14-19) to clarify this point and provide full context to the readers.

• Point 31, P12, L31-34, Discussion: The reference should be changed, as ref. 22 does not report "evidence supporting minimal handling protocols in neonatal intensive care," but merely an evaluation of replacing umbilical venous catheters with percutaneously inserted central catheters.
Response : We sincerely apologize for this citation oversight and thank the reviewer for their review. We agree that the previous reference was inappropriate and did not accurately support the statement regarding minimal handling protocols.
In accordance with your recommendation, the erroneous reference has been removed from the manuscript. The sentence has been corrected and, where appropriate, coupled with the correct bibliographical reference that explicitly addresses the neuroprotective and infectious rationale behind minimal handling practices in the NICU.

• Point 32, P12, L38, Discussion: "Infections" should be made more precise — replace with "CLABSI."
Response : In accordance with the Reviewer's guidance, we have amended this sentence in the Discussion section (Page 15) to explicitly replace "infections" with "CLABSI".

• Point 33, P12, L41, Discussion: “The reduction in infections may be attributed to ... and protecting vulnerable infants from environmental stress by moving the infusion hub outside the incubator.” This sentence should be revised, as "protection from environmental stress" has no direct impact on infection risk, but rather supports the infant's physiological stability by reducing the number of interventions and sleep interruptions. Other reasons why the CMLIS might help reduce the CLABSI rate should be presented: a closed-line system avoiding repeated connection/disconnection, tubing changes every 21 days instead of 7, etc. It should also be emphasized that this reduction in CLABSI rate was observed even though the duration of catheter use was longer than the 7 days usually recommended for changing the open multi-lumen infusion systems currently in use.
Response : We agree that protection from environmental stress and minimal handling support the neonate’s neurodevelopmental and physiological stability (such as sleep preservation) rather than exerting a direct causal effect on microbial contamination.
In accordance with your guidance, we have thoroughly revised this section of the Discussion to achieve three main objectives:
- We have explicitly detailed the structural features of the CMLIS that prevent intraluminal contamination, highlighting its pre-assembled, closed circuit design that minimizes

connection/disconnection entry points (page 15, lines 20-22).
- We have emphasized the clinical finding that a reduction in CLABSI incidence density was successfully achieved even though the CMLIS lines remained in place for up to 21 days, well beyond the traditional 7-day replacement limit standard for conventional open multi-line configurations (page 15, line 23).

• Point 34, P13, L8-15, Discussion: The reduction in CLABSI rate and antibiotic exposure is unlikely to reduce length of hospital stay or mortality in a population exposed to so many other confounding factors; it is more likely to affect outcomes related to gut microbiota and dysbiosis, such as digestive tolerance, NEC, duration of parenteral nutrition, and postnatal growth.
Response: In accordance with your comment, we have revised this section of the Discussion to explicitly state that the CMLIS had a negligible impact on mortality and NICU length of stay, clarifying that these ultimate outcomes are governed by the non-infectious complexities of prematurity rather than infectious events (page 16, line 13-17).
Furthermore, as requested in your previous comment (Point 28), we have expanded Table 1 (page 23) to include more proximate outcomes that are closely tied to gut health, dysbiosis, and clinical course, specifically NEC rates and the duration of parenteral nutrition. This adjustments helps reframe the clinical evaluation around the outcomes most likely to be affected by minimized microbial and antibiotic exposure.

• Point 35, P13, L18-22, Discussion (see point 15). The authors refined the analytical methodology, but ultimately confirmed the results already reported in reference 3. This should be discussed further. Are the differences in the magnitude of CLABSI rate reduction (-51% vs. -88%) related to differences in the statistical methods used, the study populations, the type of central venous line, the criteria used to define CLABSI, and/or the high baseline CLABSI rate (16.4/1000 catheter-days)?
Response: Delineating why the effect size in our study appears more conservative (-46.2% unadjusted, -51% adjusted reduction) compared to the initial pioneering Lyon cohort (-88%) is highly valuable for the external validity of the device.
As suggested, we have expanded our Discussion to address these divergent magnitudes (page 16, lines 1-5). The difference might be driven by two factors:
- Statistical Methodology: While the initial single-center evaluation relied on raw historical comparisons, our use of multivariable Poisson regression and Propensity Score Matching (PSM) explicitly adjusted for baseline clinical severity and neonatal risk profiles, which mathematically yields more conservative, epidemiologically realistic, and robust effect sizes.
- Baseline Institutional Factors: Variations in pre-existing, multi-component infection prevention protocols between the two tertiary centers likely influenced the "room for incremental improvement" that the introduction of a closed system could provide.
By discussing these points, we show that even when subjected to stricter statistical scrutiny and implemented within an already optimized hygiene framework, the CMLIS consistently demonstrates a highly significant and reproducible protective effect.

• Point 36, P13, L27, Discussion: Additional limitations should be acknowledged, such as the study not being powered to detect an effect on neonatal morbidities, etc.
Response : We have added this crucial point to the Limitations subsection within the Discussion (page 17, lines 17-19), explicitly acknowledging that the study was not powered to evaluate changes in secondary neonatal morbidities, total NICU length of stay, or overall mortality.

• Point 37: references should be checked and corrected.
Risposta : In accordance with the Reviewer's remarks throughout the evaluation, we have conducted a comprehensive, line-by-line audit of the entire reference list (page 19-22).
Reviewer #3
Kristina Bryant

Comments to the Author
Dacquino and colleagues describe the impact of a closed multi-lumen infusion system (CMLIS) on probable central-line associated bloodstream infections and antibiotic use in preterm neonates.

Many neonatal intensive care units continue to see CLABSIs in preterm infants despite reliable implementation of evidence-based bundles, making evaluation of new strategies and technologies important.

1) The authors refer several times to “very preterm infants.” The World Health Organization defines “very preterm” as 28 to less than 32 weeks gestation. It would be good to clarify if this is the definition intended by the authors or they are using the term more generally."
Response : The World Health Organization strictly differentiates between "extremely preterm" (<28 weeks) and "very preterm" (28 to <32 weeks) infants. In our study, we used the term "very preterm infants" in its broader clinical trial convention to define our objective inclusion thresholds, which encompass all neonates born at a gestational age of less than 32 weeks (specifically < 31 +6 weeks) and/or with a birth weight of less than 1,500 g. This means our study population dynamically includes both extremely preterm (<28 weeks) and very preterm (28 to <32 weeks) infants.
In accordance with the Reviewer’s guidance, we have updated the Population and Inclusion Criteria section within the Methods (page 6, line 13) to explicitly detail these exact gestational age and birth weight cutoffs, formally clarifying how our cohort maps onto the WHO classification to avoid any ambiguity for the reader.

2) In the Methods section, it would be beneficial to provide more details about the neonatal intensive care unit that conducted the study, including number of beds and annual admissions.
Response : In accordance with the Reviewer's request, we have expanded the Patients and Setting subsection within the Methods (page 7, lines 15-19) to provide these specific institutional details. We have clarified that our level III facility is a highly specialized unit dedicated exclusively to the care of preterm infants, completely excluding both healthy and pathological term neonates. Furthermore, we have explicitly stated that the unit operates a total of 24 beds (14 tertiary intensive care beds and 10 intermediate/sub-intensive care beds) and registered 211 total admissions during the 2024 baseline year.

3) The title and abstract of the work reference CLABSI as an outcome. In the Outcomes section, the authors clarify that the primary aim of the study is to compare the incidence of “probable CLABSI” in infants managed with standard infusion sets or CMLIS. Is “probable CLABSI” part of national infection prevention surveillance definitions in France or was this a definition adopted for this study? Was the outcome assessed by the investigators or the hospital’s infection prevention team?
Response: Regarding the definition "probable CLABSI": The term was an operational nuance originally used during our data-cleaning phase, but it does not belong to official French national surveillance definitions. To eliminate any potential ambiguity and ensure our work is fully generalizable and aligned with both national (Santé publique France / CPIAS) and international (CDC/NHSN) surveillance standards, we have completely removed the term "probable CLABSI" from the manuscript. The text, abstract, and tables now strictly and uniformly refer to standard, validated CLABSI criteria.
Regarding outcome assessment: The primary outcome was retrospectively assessed, adjudicated, and verified directly by the study investigators through a comprehensive review of clinical charts and microbiological data, rather than by the hospital's routine infection prevention team.
We have thoroughly revised the Methods (Outcomes) section (page 8, lines 3-8) to explicitly clarify both the standardized definition used and the investigator-led nature of the adjudication process.

4) The sample size calculation was based on a pre-intervention composite outcome rate. Could this composite outcome rate be defined in the paper?

Response: In tandem with addressing a related methodological critique from Reviewer 2 (Point 24) regarding our retrospective before-and-after design, we recognized that framing this section as a prospective sample size calculation based on a historical composite rate was methodologically ambiguous and inaccurate.
Because our sample size was structurally fixed by institutional annual admission volumes (a convenience sample of 170 patients), we have completely overhauled this paragraph in the revised Methods section to ensure full transparency (page 9, lines 2-12).
We have entirely removed the reference to the "composite outcome rate." Instead, we now clearly present this as a post hoc calculation of available statistical power driven directly by our true primary endpoint: the baseline historical CLABSI incidence density (15.38 per 1,000 catheter-days) evaluated under a Poisson framework. This explicitly defines the exact baseline parameters used to determine that our fixed sample size provided greater than 80% power to detect an IRR ≤ 0.50.

5) The term “probable CLABSI” should be used throughout the paper, including in the results section and tables.
Response: We understand the reviewer’s desire for terminological consistency across the manuscript. However, as detailed in our response to Point 3, rather than extending the use of the non-standard operational term "probable CLABSI," we decided that the most rigorous approach to ensure clarity and generalizability was to completely remove this term from the entire paper.
Instead, we have strictly and uniformly adopted the official CDC 2026 surveillance definitions for CLABSI across all sections of the revised manuscript, including the Abstract, Methods, Results, and Tables. By ensuring that every infection reported strictly meets these validated international criteria, we eliminate any methodological ambiguity and ensure our findings are fully transparent and directly comparable to other international neonatal cohorts.

6) The definition of probable CLABSI included a single positive blood culture. The potential that some of the patients with probable CLABSI could have actually had contaminated blood cultures should be acknowledged as a limitation.
Response: As suggested, we have transparently added this methodological vulnerability to the Limitations section of the Discussion (page 17, lines 20-24).
We have also clarified the steps taken to minimize this misclassification bias. Specifically, we applied strict, standardized CDC/NHSN clinical criteria (requiring objective clinical signs of infection for common skin commensals) coupled with a strict 5-day antibiotic treatment filter (ensuring that only cultures resulting in a full, clinically intended course of targeted therapy were counted). Crucially, because the same diagnostic protocols, clinical staff, and laboratory techniques were maintained across the study window, any residual surveillance noise from culture contamination would have affected the historical (2024) and contemporary (2025) cohorts equally, leaving the observed relative reduction unaffected.

7) It would be beneficial to know the distribution of organisms associated with positive blood cultures.
Response : We completely agree that detailing the microbiological spectrum considerably strengthens the biological plausibility of our proposed mechanism. In accordance with your suggestion, we have now included the microbiological isolates in the Results section (Page 12, lines 23-24). Specifically, we report that Coagulase-Negative Staphylococci were the predominant pathogens across both periods, which aligns with typical neonatal CLABSI etiology. Since CoNS are the primary pathogens in these device-associated infections, their reduction directly supports the hypothesis of decreased catheter hub contamination. Furthermore, this specific microbiological profile perfectly contextualizes the observed 41% decrease in vancomycin days of therapy, as vancomycin represents our cornerstone therapy for CoNS. We believe this addition significantly clarifies the antimicrobial stewardship benefits observed in our cohort
8) In the Discussion section, the authors note that a temporal effect cannot be excluded based on the retrospective nature of study. Were there any other temporal trends that could have impacted

infection rates, such as improved hand hygiene compliance or central line bundle reliability? If not, it would be good to explicitly state this.
Response: To address your query directly: at an institutional level, there were no major structural or protocol changes introduced during the study window that could account for the observed drop in infection rates. The core components of our pre-existing CLABSI prevention program, general hygiene standards, clinical workflows, and critical organizational metrics, such as nursing staffing levels and nurse-to-patient ratios, remained strictly stable across both the 2024 (control) and 2025 (CMLIS) periods. Furthermore, the implementation of the CMLIS involved structured training that focused exclusively on the technical mechanics of operating the new closed-system device, deliberately avoiding any modifications to broader infection control policies.
However, because this study is retrospective and historical, we lack granular, day-to-day prospective audit data to definitively rule out minor, unrecorded behavioral shifts (such as subtle, spontaneous fluctuations in hand hygiene compliance or line manipulation frequency) (page 17, lines 10-16).
In accordance with your valuable suggestion, we have explicitly updated the Discussion (page 16-17) to confirm the stability of our baseline institutional practices and nursing ratios. Concurrently, we have expanded our Limitations section (page 17, lines 10-16) to transparently acknowledge the absence of granular behavioral compliance data as a source of potential residual confounding.

9) A CLABSI rate of 16.42 per 1000 catheter days is relatively high. Even while acknowledging that the study was measuring probable CLABSI, this high baseline rate may limit generalizability to other NICUs.
Response: We appreciate the reviewer’s focus on the baseline infection rate and its implications for the generalizability of our findings to other neonatal intensive care units.
First, we would like to clarify a minor numerical discrepancy: as reported in our updated Table 1, the exact baseline CLABSI incidence density in our control cohort was 15.38 per 1,000 catheter-days (rather than 16.42).
We completely understand how this raw, unstratified baseline rate might initially appear elevated compared to generic international benchmarks. However, this absolute figure is an artifact of case-mix bias, driven by the exceptionally high concentration of extremely low birth weight (ELBW) admitted to our tertiary referral center.
To properly evaluate generalizability, a raw rate must be contextualized through weight-stratified data. As detailed in our expanded Discussion (and in our response to Reviewer 2, Point 30), when our baseline CLABSI incidence density is stratified by birth weight, our center’s rates are highly comparable to, and tightly aligned with, established national registry benchmarks from the French Néocat registry for non-umbilical central lines:
• Birth weight ≤750 g: Our baseline rate was 20.8 vs. 22.0 per 1,000 catheter-days in the Néocat registry.
• Birth weight 751–1000 g: Our baseline rate was 17.8 vs. 17.2 per 1,000 catheter-days in the Néocat registry.
• Birth weight 1001–1500 g: Our baseline rate was 8.7 vs. 8.4 per 1,000 catheter-days in the Néocat registry.
This precise alignment demonstrates that our baseline infection density reflects the expected, standard risk profile of a highly vulnerable, extremely preterm population rather than local practice deficiencies. Consequently, because our population matches national registry cohorts so closely when adjusted for risk, our findings regarding the efficacy of the CMLIS remain highly generalizable to other tertiary NICUs caring for similarly fragile infants.
Finally, as noted in our responses to Points 3 and 5, we have completely abandoned the non-standard term "probable CLABSI" and re-adjudicated all cases using strict, standard CDC/NHSN surveillance definitions, further reinforcing the external validity of our data.

VERSION 2 – REVIEW

**REVIEWER NAME**	van Rens, Roland

**REVIEWER AFFILIATION**	Hamad Medical Corporation, Neonatal Intensive Care Unit (NICU) Women's Wellness and Research Center
**REVIEWER CONFLICT OF INTEREST**	No Conflict of Interest
**AI DISCLOSURE**	OpenAI ChatGPT was used to assist with language editing, structuring, and clarity of the reviewer comments. The scientific assessment, critical appraisal of the manuscript, interpretation of the findings, and final recommendations were made by the reviewer.
**DATE REVIEW RETURNED**	24-Aug-2026

**GENERAL COMMENTS**	Thank you for the careful and comprehensive revision of this manuscript and for the detailed responses to my previous comments.<br>The manuscript has improved substantially. In particular, I appreciate the clearer positioning of the CMLIS as an adjunct to an established multifaceted CLABSI-prevention programme, rather than as a stand-alone solution. The additional information regarding insertion and maintenance bundles, vascular access expertise, hub disinfection, catheter securement, audit practices, and the use of cyanoacrylate tissue adhesive provides important clinical context that was lacking in the original submission.<br><br>I also appreciate the more appropriate interpretation of the historical before-and-after design. The revised manuscript now acknowledges that multivariable adjustment and propensity score matching can address measured differences but cannot eliminate residual confounding, secular trends, or other contemporaneous changes in practice. Similarly, the proposed mechanism involving reduced line manipulation is now appropriately presented as biologically plausible rather than demonstrated, since manipulation frequency, catheter access, hub contamination and workflow were not prospectively measured.<br><br>Most of my previous concerns have therefore been satisfactorily addressed. I have only a few remaining points that I believe should be clarified before publication.<br><br>1. CLABSI definition and the ≥5-day antibiotic criterion<br>I appreciate the removal of the term “probable CLABSI” and the clearer reference to CDC/NHSN criteria. However, the manuscript also applies an additional requirement of at least five days of targeted antimicrobial treatment. The authors themselves appropriately acknowledge that this additional criterion may influence case classification.<br>I would recommend making an even clearer distinction between the established surveillance definition and the study-specific modification. Rather than implying that the outcome represents the unmodified standard CDC/NHSN definition, please state explicitly that CLABSI was classified according to CDC/NHSN criteria with an additional study-specific ≥5-day treatment criterion, if that accurately reflects the methodology.<br>This distinction is particularly relevant because antibiotic exposure is itself a secondary outcome of the study. Treatment decisions therefore contribute to the classification of the primary outcome while antibiotic treatment is simultaneously evaluated as an outcome.<br><br>2. Derived number needed to treat<br>I remain uncertain about the clinical interpretation of the reported

	NNT of approximately 8. The primary outcome is an incidence density expressed per 1,000 catheter-days, and the NNT is subsequently derived using the observed average catheter duration. The manuscript states that the adjusted incidence rate difference of −6.5/1,000 catheter-days translates, using a mean catheter duration of 19.12 days, to approximately eight patients treated to prevent one CLABSI.<br>A conventional NNT is generally based on an absolute patient-level risk difference over a defined follow-up period. Deriving an NNT from an incidence-rate difference requires assumptions regarding exposure duration and constant event rates and may therefore give readers a stronger impression of a patient-level treatment effect than is justified by these data.<br>I suggest either providing a clear methodological justification for this conversion and qualifying its interpretation, or, preferably, removing the NNT and retaining the adjusted incidence rate difference per 1,000 catheter-days, which is already clinically meaningful and directly reflects the study design.<br><br>3. Conclusion and implications for practice<br>The revised Discussion is considerably more balanced. However, I would still slightly temper the conclusion that the findings support “integration of the CMLIS into routine line management and antimicrobial stewardship.” Given the retrospective single-centre historical comparison and the acknowledged possibility of residual temporal confounding, I believe this goes somewhat beyond what the data can establish.<br>I would suggest wording that emphasizes that the findings support further prospective evaluation of CMLIS as an adjunct to established CLABSI-prevention strategies, rather than directly supporting routine implementation. This would also align better with the authors' own appropriate statement that prospective studies are required.<br><br>Minor terminology comment<br>The manuscript continues to use the term “epicutaneo-caval catheter (ECC)”. For an international readership, I would encourage the authors to consider internationally recognizable vascular access terminology, particularly neonatal peripherally inserted central catheter (n-PICC) where this accurately describes the device and tip location. The locally used terminology could, if necessary, be provided at first mention. The important point is to make clear that these are central vascular access devices and to use the terminology consistently throughout.<br><br>Overall assessment<br>This is now a substantially stronger manuscript. The authors have responded constructively to the previous review and, importantly, have appropriately reframed the CMLIS as one component of a broader infection-prevention strategy rather than attributing the observed CLABSI reduction solely to the device.<br>The remaining issues relate primarily to precision of outcome definition, interpretation of the derived NNT, and the strength of the clinical recommendation. These should be addressable without substantial additional analysis.

VERSION 2 – AUTHOR RESPONSE

Reviewer: 1
Mr. Roland van Rens, Hamad Medical Corporation
Comments to the Author
Thank you for the careful and comprehensive revision of this manuscript and for the detailed responses to my previous comments.
The manuscript has improved substantially. In particular, I appreciate the clearer positioning of the CMLIS as an adjunct to an established multifaceted CLABSI-prevention programme, rather than as a stand-alone solution. The additional information regarding insertion and maintenance bundles, vascular access expertise, hub disinfection, catheter securement, audit practices, and the use of cyanoacrylate tissue adhesive provides important clinical context that was lacking in the original submission.

I also appreciate the more appropriate interpretation of the historical before-and-after design. The revised manuscript now acknowledges that multivariable adjustment and propensity score matching can address measured differences but cannot eliminate residual confounding, secular trends, or other contemporaneous changes in practice. Similarly, the proposed mechanism involving reduced line manipulation is now appropriately presented as biologically plausible rather than demonstrated, since manipulation frequency, catheter access, hub contamination and workflow were not prospectively measured.

Most of my previous concerns have therefore been satisfactorily addressed. I have only a few remaining points that I believe should be clarified before publication.

1. CLABSI definition and the ≥5-day antibiotic criterion
I appreciate the removal of the term “probable CLABSI” and the clearer reference to CDC/NHSN criteria. However, the manuscript also applies an additional requirement of at least five days of targeted antimicrobial treatment. The authors themselves appropriately acknowledge that this additional criterion may influence case classification.
I would recommend making an even clearer distinction between the established surveillance definition and the study-specific modification. Rather than implying that the outcome represents the unmodified standard CDC/NHSN definition, please state explicitly that CLABSI was classified according to CDC/NHSN criteria with an additional study-specific ≥5-day treatment criterion, if that accurately reflects the methodology.
This distinction is particularly relevant because antibiotic exposure is itself a secondary outcome of the study. Treatment decisions therefore contribute to the classification of the primary outcome while antibiotic treatment is simultaneously evaluated as an outcome.

Response : We thank the reviewer for this methodological observation. We fully agree that distinguishing the standard surveillance definition from our study-specific criterion is essential, especially given the inherent interrelation between treatment decisions and our primary and secondary outcomes. In accordance with your recommendation, we have made the following revisions to the manuscript:
- Methods (page 8, lines 8-11) We have explicitly reformulated the definition to distinguish the established CDC/NHSN criteria from our additional study-specific requirement.
- Discussion (page 17, lines 20-25): We have transparently addressed this limitation, noting that the ≥5-day treatment filter inherently links the classification of the primary outcome (CLABSI) to our secondary outcome (antibiotic exposure).

2. Derived number needed to treat
I remain uncertain about the clinical interpretation of the reported NNT of approximately 8. The primary outcome is an incidence density expressed per 1,000 catheter-days, and the NNT is

subsequently derived using the observed average catheter duration. The manuscript states that the adjusted incidence rate difference of −6.5/1,000 catheter-days translates, using a mean catheter duration of 19.12 days, to approximately eight patients treated to prevent one CLABSI.
A conventional NNT is generally based on an absolute patient-level risk difference over a defined follow-up period. Deriving an NNT from an incidence-rate difference requires assumptions regarding exposure duration and constant event rates and may therefore give readers a stronger impression of a patient-level treatment effect than is justified by these data.
I suggest either providing a clear methodological justification for this conversion and qualifying its interpretation, or, preferably, removing the NNT and retaining the adjusted incidence rate difference per 1,000 catheter-days, which is already clinically meaningful and directly reflects the study design.
Response : We completely agree with your epidemiological assessment. We acknowledge that deriving a NNT from an incidence rate difference relies on strong assumptions regarding uniform exposure duration and constant event rates. As you rightly pointed out, this conversion risks inadvertently overstating a binary, patient-level treatment effect in a preterm neonatal cohort characterized by highly variable catheter dwell times.
Following your recommendation, we have removed the NNT calculation entirely from the Results, Discussion, and Table 3. We now rely exclusively on the adjusted incidence rate difference per 1,000 catheter-days, which directly and robustly reflects our study design and multivariable Poisson regression methodology without requiring unwarranted mathematical assumptions.

3. Conclusion and implications for practice
The revised Discussion is considerably more balanced. However, I would still slightly temper the conclusion that the findings support “integration of the CMLIS into routine line management and antimicrobial stewardship.” Given the retrospective single-centre historical comparison and the acknowledged possibility of residual temporal confounding, I believe this goes somewhat beyond what the data can establish.
I would suggest wording that emphasizes that the findings support further prospective evaluation of CMLIS as an adjunct to established CLABSI-prevention strategies, rather than directly supporting routine implementation. This would also align better with the authors' own appropriate statement that prospective studies are required.
Response: We thank the reviewer for this feedback. We completely agree that the retrospective, single-center nature of our study, along with the inherent risk of residual temporal confounding, precludes making direct recommendations for routine clinical implementation.
We have carefully revised the Conclusion to temper our statement (page 18, lines 3-7). We removed the suggestion for routine integration and instead emphasized that our findings support the need for further prospective evaluation of the CMLIS as an adjunctive measure to established CLABSI-prevention and antimicrobial stewardship strategies.

Minor terminology comment
The manuscript continues to use the term “epicutaneo-caval catheter (ECC)”. For an international readership, I would encourage the authors to consider internationally recognizable vascular access terminology, particularly neonatal peripherally inserted central catheter (n-PICC) where this accurately describes the device and tip location. The locally used terminology could, if necessary, be provided at first mention. The important point is to make clear that these are central vascular access devices and to use the terminology consistently throughout.
Response : We fully agree that adopting standardized, internationally recognized terminology greatly improves the manuscript's clarity and accessibility for a global readership. We have removed all instances of the local term "epicutaneo-caval catheter (ECC)" and replaced them exclusively with "neonatal peripherally inserted central catheter (nPICC)" consistently throughout the text, tables, and figures to clearly denote central vascular access (page 6 line 15, page 7 line 13).

Overall assessment
This is now a substantially stronger manuscript. The authors have responded constructively to the previous review and, importantly, have appropriately reframed the CMLIS as one component of a broader infection-prevention strategy rather than attributing the observed CLABSI reduction solely to the device.
The remaining issues relate primarily to precision of outcome definition, interpretation of the derived NNT, and the strength of the clinical recommendation. These should be addressable without substantial additional analysis.

We wish to express our sincere gratitude to the Mr. Roland van Rens for the time, patience, and meticulous care dedicated to evaluating our manuscript. His insightful methodological critiques have not only significantly improved the rigor and clarity of this paper but have also provided a profoundly valuable learning experience for our team. We are deeply appreciative of his constructive guidance.
